# Protocol for the SACLA trial: Efficacy and safety of subretinal monteplase for submacular hemorrhage in a phase II single-arm multicenter decentralized clinical trial

**DOI:** 10.1371/journal.pone.0353127

**Published:** 2026-07-02

**Authors:** Noriko Yoshida, Yuko Kobayakawa, Kouta Funakoshi, Sakiko Kimura, Kazuhisa Hosoya, Yuya Shinkawa, Takuma Furukawa, Tsubasa Mitsutake, Toshimi Hoshiko, Ayako Takamori, Naoto Kawahara, Hiroto Terasaki, Akio Oishi, Hideki Koizumi, Toshihiro Inoue, Atsunobu Takeda, Yasuhiro Ikeda, Shigeo Yoshida, Yuki Morizane, Kazuaki Kadonosono, Makoto Inoue, Motohiro Kamei, Koji Todaka, Koh-Hei Sonoda, Hiroshi Enaida

**Affiliations:** 1 Clinical Research Center, Saga University Hospital, Saga, Japan; 2 Center for Clinical and Translational Research, Kyushu University Hospital, Fukuoka, Japan; 3 Department of Pharmacy, Saga University Hospital, Saga, Japan; 4 Department of Preventive Medicine, Faculty of Medicine, Saga University, Saga, Japan; 5 Education and Research Centre for Community Medicine, Faculty of Medicine, Saga University, Saga, Japan; 6 Department of Medical Technology and Science, Faculty of Fukuoka Health Care, International University of Health and Welfare, Fukuoka, Japan; 7 Department of Hematology and Oncology, Saga University Hospital, Saga, Japan; 8 Clinical Research Center in Hiroshima, Hiroshima University Hospital, Hiroshima, Japan; 9 Department of Ophthalmology, Kagoshima University Graduate School of Medical and Dental Sciences, Kagoshima, Japan; 10 Department of Ophthalmology and Visual Sciences, Graduate School of Biomedical Sciences, Nagasaki University, Nagasaki, Japan; 11 Department of Ophthalmology, Graduate School of Medicine, University of the Ryukyus, Okinawa, Japan; 12 Department of Ophthalmology, Faculty of Life Sciences, Kumamoto University, Kumamoto, Japan; 13 Department of Ophthalmology, Faculty of Medicine, Oita University, Ufu, Oita, Japan; 14 Department of Ophthalmology, Faculty of Medicine, University of Miyazaki, Miyazaki, Japan; 15 Department of Ophthalmology, Kurume University School of Medicine, Kurume, Fukuoka, Japan; 16 Department of Ophthalmology, Graduate School of Medicine, Dentistry and Pharmaceutical Sciences, Okayama University, Okayama, Japan; 17 Department of Ophthalmology, Yokohama City University Medical Center, Yokohama, Japan; 18 Kyorin Eye Center, Kyorin University School of Medicine, Mitaka, Japan; 19 Department of Ophthalmology, Aichi Medical University, Nagakute, Japan; 20 Department of Ophthalmology, Graduate School of Medical Science, Kyushu University, Fukuoka, Japan; 21 Department of Ophthalmology, Faculty of Medicine, Saga University, Saga, Japan; PLOS: Public Library of Science, UNITED STATES OF AMERICA

## Abstract

Submacular hemorrhage (SMH), which may arise from age-related macular degeneration, retinal arterial macroaneurysm, and other causes, can result in severe vision loss and central visual field impairment. Although tissue plasminogen activator (tPA) is used off-label to treat SMH in many countries, no formulation has been approved for this indication. Because early intervention is critical when tPA is used for SMH, limited access to centers that can provide this treatment may delay care and reduce treatment opportunities. Decentralized clinical trials (DCTs) reduce or eliminate the need for participants to travel to trial sites. We therefore designed the investigator-initiated SACLA trial to evaluate subretinal tPA for SMH. The DCT framework is intended to reduce logistical barriers related to the disease severity and rarity. The SACLA trial is a phase II multicenter, open-label, single-arm surgical study with a pre-post comparison design (jRCT2071250003). Twenty eligible participants will undergo pars plana vitrectomy followed by subretinal injection of 0.1 mL (8,000 IU) of tPA. Participants will remain hospitalized at the trial site until the primary outcome, change in central foveal thickness (CFT) from baseline at Week 1, is assessed. Thereafter, follow-up visits will be conducted at either the trial site or partner sites within the DCT framework. Secondary efficacy outcomes include change in CFT from baseline, presence of a foveal hemorrhage measuring at least 1 disc diameter, best-corrected visual acuity (BCVA), and change in BCVA from baseline at Weeks 4 and 12. Adverse events will be collected throughout the 12-week observation period to assess safety. The protocol and related study documents were reviewed and approved by the Saga University Hospital Institutional Review Board. This study is designed to generate prospective evidence on the feasibility, short-term anatomical response, and safety of subretinal tPA for SMH.

## Introduction

Submacular hemorrhage (SMH) is characterized by blood accumulation beneath the retina, most commonly secondary to age-related macular degeneration (AMD) and rupture of retinal arterial macroaneurysms (RAM) [1,2]. SMH causes retinal damage through toxic, tractional, and barrier effects [3]. It also causes acute, severe visual impairment, and more than 80% of patients have a final visual acuity of <0.1 without treatment [4]. The annual incidence of SMH larger than the optic disc is reportedly 5.4–25 cases per million people, indicating that it is a rare disease [5,6].

If SMH becomes chronic, it can lead to irreversible neuroretinal degeneration and poor visual outcomes [7–9]. Therefore, the goal of SMH treatment is to displace the hemorrhage away from the macula, or at least the fovea, as quickly as possible [2]. Because SMH varies in cause, location, volume, and duration of bleeding, clear treatment guidelines have not yet been established. Treatment is selected according to the underlying disease and bleeding characteristics and may include intravitreal anti-vascular endothelial growth factor (VEGF) injection for choroidal neovascularization, pneumatic displacement with intravitreal gas, subretinal hematoma removal, or retinal photocoagulation [10–13].

Tissue plasminogen activator (tPA) is a key fibrinolytic enzyme that converts plasminogen to plasmin and thereby promotes thrombolysis. Intravitreal or subretinal tPA has been suggested to reduce fibrin-mediated damage to photoreceptors and the retinal pigment epithelium (RPE). In addition, combined use with vitrectomy or intraocular gas may facilitate clot displacement and help prevent vision loss [14–16]. In Japan, several studies have reported the efficacy and safety of local tPA treatment for SMH associated with AMD or RAM [17–29]. However, tPA has not been approved for SMH and therefore continues to be used off-label worldwide. Consequently, the number of facilities able to administer tPA remains limited. Moreover, tPA is currently the only available agent for promoting submacular hematoma displacement. As a result, patients may need to delay or forgo treatment because visual impairment can make travel to distant facilities difficult.

Decentralized clinical trials (DCTs) use digital and other innovative technologies to reduce or eliminate the need for patients to visit the trial site (investigational site) where the principal investigator (PI) or sub-investigator (SI) is based [30–33]. With appropriate planning, protocol design, risk-based assessment, and regulatory engagement, DCTs may support a more patient-centric approach to medical research, particularly in rare diseases [34]. Studies from Japan have reported implementation of DCTs using digital technologies such as telemedicine, particularly in oncology [35]. In addition, certain clinical trial-related tasks may legally be delegated to local hospitals or clinics designated as partner sites in addition to the trial site [36]. Such collaboration may facilitate more patient-centered clinical trials. However, the use of partner sites in acute conditions has rarely been reported, likely because rapid intervention requires a well-coordinated care system.

In June 2016, the National Institutes of Health (NIH) released a policy on the use of a single institutional review board (IRB) for multisite research to improve trial efficiency and participant protection [37]. The benefits of a single IRB in making multisite research faster and more efficient are expected to outweigh the burden associated with changing traditional review processes [38]. In the Japanese regulatory framework, academic research is divided into four categories, and investigator-initiated clinical trials intended for regulatory approval are conducted under the Pharmaceuticals and Medical Devices Act (PMD Act) [39]. Ethical review of multisite trials, particularly investigator-initiated clinical trials, has traditionally been conducted by the IRB at each participating institution [40]. This duplication can increase administrative burden and reduce efficiency, highlighting the need for a single-IRB review system.

We therefore designed a phase II investigator-initiated clinical trial using a DCT framework to evaluate the efficacy and safety of subretinal tPA for SMH and to support future indication expansion or ophthalmic formulation development. Use of a DCT framework may help address logistical challenges related to disease severity and rarity. In addition, adoption of a single IRB may improve review efficiency, shorten the time to trial initiation, and reduce the administrative burden of trial management.

## Methods and analysis

### Study design

This multicenter, open-label, single-arm, investigator-initiated phase II trial will evaluate the safety and efficacy of monteplase (genetical recombination; Eisai Co, Tokyo, Japan) in patients with SMH associated with AMD or RAM. This trial will enroll patients with severe disease, defined by a large hematoma unlikely to resolve spontaneously, foveal involvement, and anticipated substantial vision loss. A pre-post comparison will be used to assess objective measures of hematoma burden and visual function before and after tPA administration. A placebo control was not adopted because it would offer no therapeutic benefit and could expose patients to procedure-related risk. Accordingly, this study was designed as an open-label, single-arm trial without a control group.

### Study setting and decentralized framework

**Fig 1:**
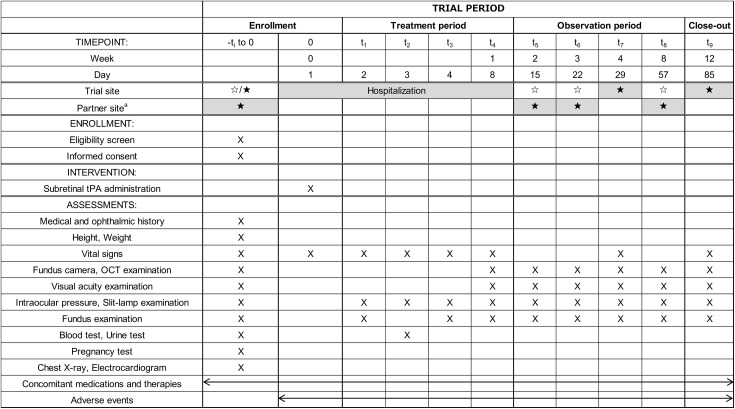
Participant timeline: Schedule of enrollment, intervention, and assessments. ^a^ If there is no partner site, the participant will visit the trial site. ☆ Telemedicine, ★ In-person visit. OCT: optical coherence tomography.

The trial will be conducted at nine trial sites (investigational sites) in Japan, supported by a network of partner sites involved in participant referral and follow-up. Participants will be recruited through digital media, including websites targeting medical professionals, using an electronic recruitment (eRecruitment) strategy. Some trial visits may be conducted at partner sites that meet the criteria listed in Fig 1.

However, even when a visit is conducted at a partner site, the PI or SI at the trial site will remain responsible for trial-related activities. Institutions will be selected as partner sites if they meet all of the following criteria: (1) availability of at least one physician certified by the Japanese Ophthalmological Society; (2) regular inspection and quality control of equipment used for trial observations; and (3) availability of internet access and telemedicine equipment, including a webcam with sufficient resolution to confirm test results, to connect the partner site with the trial site.

**Fig 2:**
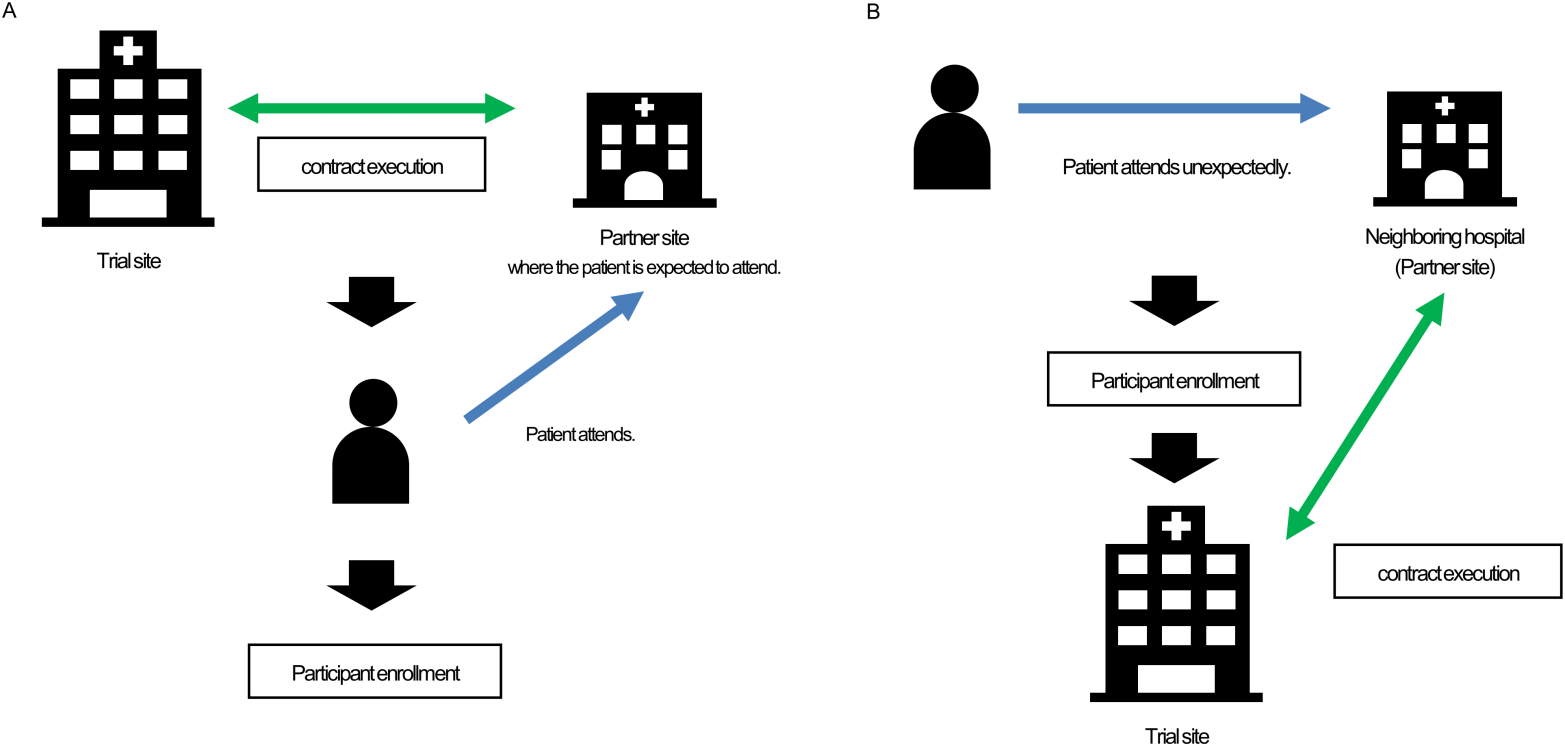
Clinical trial contract models for partner sites. **(A)** Standard clinical trial contract: The trial site executes a contract with a neighboring hospital where a patient with SMH is expected to attend in order to delegate specific trial-related duties to the partner site. The participant is then enrolled in this trial. **(B)** Dynamic partnering: Because it is difficult to predict when and where patients with SMH will develop symptoms, they may present to unexpected medical facilities. If the referring neighboring hospital meets the required criteria, a contract is established with that hospital as a partner site after participant enrollment.

The trial site will execute a contract with each partner site. In this study, “dynamic partnering” is defined as contract execution, after participant enrollment, with a neighboring hospital or clinic that referred the patient and meets the criteria for a partner site (Fig 2).

The following procedures may be performed when participants visit partner sites: explanation of the trial and obtaining electronic consent (eConsent); screening assessments; scheduled evaluations at Visit 6 (Week 2/Day 15), Visit 7 (Week 3/Day 22), and Visit 9 (Week 8/Day 57); evaluation at treatment discontinuation; and unscheduled visits, if deemed appropriate by the PI or SI.

On the day of the visit, the trial site and partner site will be connected through the telemedicine system, and test results obtained at the partner site will be reviewed by the PI or SI at the trial site via webcam. After completion of the remote consultation, the physician at the partner site will date and sign the printed test results and mail them to the trial site as certified copies.

At the time of protocol publication, partner sites include NAKAHARA EYE CLINIC, NOGUCHI EYE CLINIC, MIKAWA EYE CLINIC, SAGA-KEN MEDICAL CENTRE KOSEIKAN, ASO IIZUKA HOSPITAL, Sakisaka Eye Clinic, and iryouhoujin Yozankai Igoganka. Additional partner sites may be added following the required regulatory and ethical approvals.

### Eligibility criteria

After informed consent is obtained, potential participants will be screened by ophthalmologists according to the following criteria:

#### Inclusion criteria.

**Age:**

1) Age *>* 18 years at the time of informed consent.

**Condition:**

2) SMH involving the fovea in the study eye secondary to AMD or RAM.
3) Central foveal thickness (CFT) > 300 µm in the study eye as measured by optical coherence tomography (OCT) at screening.
4) Best-corrected visual acuity (BCVA) <0.2 (decimal notation) in the study eye at screening.

**Other:**

5) Ability to provide written consent; if written consent cannot be confirmed because of visual impairment, oral consent will be documented in the presence of a witness and handled in accordance with IRB-approved procedures.

#### Exclusion criteria.

Medical conditions

1) Significant organization or fibrosis due to prolonged SMH, as determined by the PI or SI at screening.
2) Coexisting active proliferative diabetic retinopathy, uveitis, or optic neuritis at screening.
3) Ocular complications, such as macular atrophy or fibrosis, that are judged by the PI or SI at screening to limit the likelihood of visual improvement.

Factors affecting trial intervention and evaluation

4) Anterior segment or vitreous abnormalities at screening that interfere with fundus evaluation by OCT, color fundus photography, or fluorescein angiography.
5) Known allergy to any component of the investigational drug or a history of such allergy.
6) Alcohol or drug dependence, or psychiatric disorders that may interfere with study participation.
7) Planned ophthalmic surgery other than administration of the investigational intervention during the study period.

Factors affecting safety

8) Active bleeding, including gastrointestinal bleeding, urinary tract bleeding, retroperitoneal bleeding, intracranial hemorrhage, or hemoptysis.
9) Intracranial or spinal surgery or injury within 2 months before informed consent.
10) Intracranial tumors, arteriovenous malformations, or aneurysms.
11) PT-INR ≥ 3.1 in patients aged <70 years or ≥2.7 in patients aged ≥70 years, unless the value is expected to decrease before vitrectomy; in such cases, PT-INR must be confirmed to be < 2.7 on the day of surgery in view of systemic and intraocular bleeding risk.
12) Hypertension with systolic blood pressure remaining ≥180 mmHg despite antihypertensive treatment.
13) Women of childbearing potential not using appropriate contraception, women who are pregnant or may be pregnant, breastfeeding women, and patients planning pregnancy during the study period.

Others

14) Current participation in another clinical trial or participation in another clinical trial within 6 months before informed consent.
15) Any condition judged by the PI or SI to make the patient inappropriate for study participation.

### Interventions

#### Investigational medical product.

Monteplase is a glycoprotein produced from a gene encoding a modified human tPA derivative in which the cysteine residue at position 84 from the N-terminus is replaced with serine. It exerts thrombolytic effects by converting plasminogen within thrombi to plasmin. In Japan, it is approved for intravenous administration for dissolution of coronary artery thrombi in acute myocardial infarction and pulmonary artery thrombi in acute pulmonary embolism.

#### tPA dose.

Monteplase will be reconstituted in 5 mL of saline to yield a final concentration of 80,000 IU/mL, with care taken to ensure complete dissolution without bubble formation. A total of 0.1 mL (8,000 IU) of this solution will then be administered by subretinal injection following pars plana vitrectomy. However, if administration of the full dose is not feasible because of fundus conditions, the surgeon may adjust the injection volume at their discretion and document the administered dose.

#### Surgical technique.

The intervention procedures will be performed by experienced ophthalmologists trained in subretinal injection techniques and the management of SMH. Protocol compliance will be assessed through review of study documentation and case report forms.

1. Local or general anesthesia will be administered.
2. Vitrectomy will be performed using a 3-port system with 23-, 25-, or 27-gauge instruments.
3. Phacoemulsification and intraocular lens implantation may be performed at the discretion of the surgeon in consultation with the participant.
4. Peeling of the epiretinal membrane (ERM) or internal limiting membrane (ILM) will be permitted.
5. Monteplase will be administered with a subretinal injection needle into areas of subretinal hemorrhage away from the fovea. Areas with pigment epithelial detachment (PED) will be avoided to reduce the risk of sub-retinal pigment epithelial injection and retinal pigment epithelial rupture. Administration from multiple sites will be permitted if necessary. During injection, intraocular pressure will be monitored by palpation.
6. If a retinal tear is present, it will be treated with retinal photocoagulation or cryotherapy.
7. Gas tamponade or silicone oil tamponade will be performed, with the choice determined by the surgeon according to disease severity and the feasibility of postoperative positioning.
8. After monteplase administration, the participant will remain in the supine or prone position for 20–60 minutes, including intraoperative time, depending on the degree of bleeding. Thereafter, postoperative positioning will follow the surgeon’s instructions, and the details will be recorded in the case report form.

#### Rescue treatment.

If deemed necessary by the PI or SI, the following rescue treatments may be administered:

- Anti-VEGF therapy
- Intravitreal gas injection
- Photodynamic therapy

#### Relevant concomitant care prohibited or permitted during the trial.

The following medications and therapies will be prohibited during the trial period:

1) Defibrotide sodium (Defitelio®).
2) Intravenous, subretinal, or intravitreal administration of tPA products other than monteplase.
3) Intravenous or intravitreal administration of monteplase.
4) Ophthalmic surgical procedures other than those specified below.

The following surgical procedures and medications will be permitted during the trial period:

1) Phacoemulsification and intraocular lens implantation.
2) ERM peeling and ILM peeling.
3) Retinal photocoagulation or cryotherapy for retinal tears.
4) Antiplatelet agents (including drugs with platelet aggregation inhibitory effects) and anticoagulants.

#### Stop criteria.

If the PI or SI determines before surgery that subretinal administration of monteplase is not feasible because of deterioration in the participant’s general condition, the investigational intervention will be discontinued.

Participation in the trial will be discontinued under any of the following circumstances: (1) withdrawal of consent, with the reason documented in the case report form; (2) determination of ineligibility after enrollment; (3) investigator judgment that continuation is not appropriate based on an overall safety assessment, including the occurrence of adverse events, with the reason documented in the case report form; (4) death of the participant; (5) inability to undergo required examinations or observations because of personal circumstances after trial initiation; (6) classification as untraceable, defined as failure to attend a scheduled visit and inability to contact the participant through the trial site; or (7) any other reason judged by the PI or SI to preclude continued participation.

If treatment is discontinued, appropriate medical care will be provided at the physician’s discretion. Whenever possible, protocol-specified observations and tests will also be performed at the time of discontinuation to support safety evaluation. Participants may withdraw from the trial at any time for any reason without disadvantage. Data collected before withdrawal of consent will, in principle, be included in the analysis. No imputation will be performed unless otherwise specified.

### Outcomes and estimands

#### Primary outcome.

Change in CFT from baseline at Week 1.

#### Estimand for the primary outcome.

The primary clinical question is the extent of change in SMH at Week 1 after subretinal administration of monteplase in patients with SMH associated with AMD or RAM, regardless of concomitant anti-VEGF therapy or use of tPA formulations for other indications.

The primary estimand is defined as follows:

- Population: patients with SMH associated with AMD or RAM who meet the eligibility criteria.
- Treatment condition: subretinal administration of monteplase.
- Variable: change from baseline in OCT-measured CFT at Week 1.
- Intercurrent events: handled according to prespecified strategies (treatment policy, hypothetical, or composite).
- Summary measure: mean change from baseline.

The rationale for the primary estimand is as follows. Subfoveal hemorrhage thickness is known to affect visual prognosis in SMH. OCT-measured CFT, defined as the distance from the upper edge of RPE layer to the internal limiting membrane, reflects the combined thickness of the neurosensory retina and subfoveal hemorrhage and also permits assessment of associated hemorrhagic PED, which is related to visual outcomes. Therefore, CFT was selected as the variable for the primary estimand.

Early reduction in CFT is considered clinically important for regulatory evaluation and treatment decision-making, even when the effects of concomitant anti-VEGF therapy or other tPA formulations after monteplase administration are taken into account. Because persistent SMH adversely affects visual prognosis, early resolution is critical. Therefore, Week 1 was selected as the primary evaluation time point because CFT can be assessed before it is substantially influenced by intraocular gas tamponade used during vitrectomy.

#### Secondary efficacy outcomes.

1. Change in CFT from baseline at Week 4.
2. Presence or absence of a foveal hemorrhage measuring ≥1 disc diameter at Week 4.
3. BCVA (logMAR equivalent) at Week 4.
4. Change in BCVA (logMAR equivalent) from baseline at Week 4.
5. Change in CFT from baseline at Week 12.
6. BCVA (logMAR equivalent) at Week 12.
7. Change in BCVA (logMAR equivalent) from baseline at Week 12.

#### Estimands for secondary efficacy outcomes.

The clinical question of interest is the extent of change in ophthalmic findings at Weeks 4 and 12 after subretinal administration of monteplase in patients with SMH associated with AMD or RAM, regardless of concomitant anti-VEGF therapy or use of tPA formulations for other indications. The rationale for the secondary estimand is that SMH can cause rapid, severe visual impairment and, if prolonged, may lead to irreversible neuroretinal degeneration, thereby affecting visual prognosis. Even when the effects of anti-VEGF therapy or other tPA formulations after monteplase administration are considered, hematoma reduction, hematoma displacement, and visual recovery remain clinically important for regulatory evaluation and treatment decision-making. Although assessment of visual recovery requires long-term follow-up, changes in CFT over longer periods may be substantially influenced by factors other than subretinal tPA administration, such as spontaneous hematoma absorption and rebleeding. Therefore, Weeks 4 and 12 were selected as evaluation time points for the secondary estimand.

#### Secondary safety outcomes.

The incidence of adverse events (AEs) and adverse drug reactions (ADRs) will be evaluated through Week 12. An AE is defined as any unfavorable and unintended sign, including an abnormal clinical laboratory value, that occurs in temporal association with use of the investigational drug, regardless of whether it is considered causally related. An ADR is defined as an AE with a causal relationship to the investigational drug.

### Sample size calculation

We retrospectively reviewed 8 cases (11 eyes) of SMH treated with tPA at our hospital over the previous 2 years, limited to eyes with a pretreatment CFT of ≥300 μm. At 1 week after surgery, the mean change in CFT was −109.36, with a standard deviation of 176.98. Based on these data, the effect size (d = Mdiff/SD) was 0.62. Because SMH is a rare disease and this study targets patients with moderate to severe hemorrhage and vision loss, we assumed a modest treatment effect. Assuming an effect size of 0.7 (mean change, −120; standard deviation, 170), the required sample size was 19 cases at a two-sided significance level of 5% and 80% power. Allowing for a 5% dropout rate, the target sample size was set at 20 cases. This sample size is also expected to provide a clinically meaningful estimate of early anatomical response with acceptable precision.

### Data collection and management

Trial data will be collected using standardized case report forms by trained investigators. Ophthalmic examinations, including BCVA, fundus examination, and OCT, and laboratory tests will be performed according to routine clinical practice. Data will be entered into a validated electronic data capture (EDC) system. Data accuracy will be ensured through regular review and range checks. All study data will be anonymized and securely stored with restricted access.

### Statistical analysis

Efficacy analyses will be performed in the full analysis set (FAS), excluding participants without post-baseline efficacy data or with major protocol violations.

For the primary endpoint, the mean change from baseline in CFT at Week 1 will be summarized with a 95% confidence interval, and a one-sample t-test will be used for statistical inference. As a sensitivity analysis, the Wilcoxon signed-rank test will be used to assess robustness to potential deviations from normality.

Intercurrent events will be handled as follows. First, a treatment-policy strategy will be applied for vitreous reoperation for ocular complications, sub-Tenon triamcinolone acetonide administration, rescue photodynamic therapy or VEGF inhibitor therapy, and use of prohibited concomitant therapies such as defibrotide sodium or tPA (including monteplase) for other diseases; for these events, all observed primary-endpoint values will be included in the analysis. Second, a hypothetical strategy will be applied for consent withdrawal or loss to follow-up not due to an SAE, rescue intravitreal gas injection, and use of prohibited concomitant tPA (including monteplase) as additional treatment for SMH; for these events, data collected after occurrence of the intercurrent event will be excluded. Third, a composite strategy will be applied for vitreous reoperation due to rebleeding, withdrawal due to an SAE, and hematoma removal surgery as prohibited concomitant therapy; in these cases, the primary endpoint will be assigned a change from baseline of 0 as the worst value.

In addition to the primary and sensitivity analyses, the frequency of repeated intercurrent events or serious adverse events will be summarized, and their relationship with the primary endpoint will be explored. An additional analysis will evaluate the efficacy of the investigational drug under a hypothetical strategy that excludes the effects of anti-VEGF therapy and use of monteplase or other tPA for other diseases, which are treated as intercurrent events. This analysis is intended to support clinical interpretation at the individual-patient level. Specifically, this analysis will be conducted in the same manner as the primary analysis, excluding data collected after occurrence of the intercurrent event.

Analyses supporting the secondary outcomes will be conducted as follows:

1. For CFT at Week 4, the mean change from baseline will be summarized with a 95% confidence interval, and a one-sample t-test will be conducted.
2. For the presence of a foveal hemorrhage measuring ≥1 disc diameter at Week 4, the proportion will be summarized with exact confidence intervals, and a binomial test will be performed for exploratory purposes.
3. For BCVA (logMAR equivalent) at Week 4, the mean change from baseline will be summarized with a 95% confidence interval.
4. For BCVA (logMAR equivalent) at Week 4, the mean change from baseline will be summarized with a 95% confidence interval, and a one-sample t-test will be conducted.
5. For CFT at Week 12, the mean change from baseline will be summarized with a 95% confidence interval, and a one-sample t-test will be conducted.
6. For BCVA (logMAR equivalent) at Week 12, the mean change from baseline will be summarized with a 95% confidence interval.
7. For BCVA (logMAR equivalent) at Week 12, the mean change from baseline will be summarized with a 95% confidence interval, and a one-sample t-test will be conducted.

No formal adjustment for multiplicity will be applied to secondary outcomes, which will therefore be interpreted as exploratory. Safety analyses will summarize the number of participants, the number with events, and the incidence proportion for each safety outcome.

No interim analysis is planned for this trial because of the relatively small sample size and short study duration.

### Monitoring

#### Data monitoring committee.

No formal data monitoring committee, steering committee, or endpoint adjudication committee will be established because this is an investigator-initiated study with a limited sample size and relatively low-risk intervention.

#### AE and SAE reporting.

The PI or SI will be responsible for identifying, documenting, and reporting events that meet the definitions of an AE or serious adverse event (SAE). An AE will be classified as an SAE in the following cases: (1) death; (2) an event that is life-threatening; (3) permanent disability; or (4) hospitalization or prolongation of hospitalization. The PI or SI will also be responsible for follow-up of all AEs, including those reported by participants.

#### Trial monitoring.

Monitoring will be conducted in accordance with the trial-specific monitoring procedures to ensure compliance with Good Clinical Practice (GCP), the trial protocol, standard operating procedures, and applicable regulations, as well as to ensure data reliability. Monitoring activities will include on-site and off-site monitoring, including source data verification through comparison of source documents and case report forms. Any discrepancies identified between source documents and case report forms will be documented and clarified by the principal investigator.

### Ethics and dissemination

This trial will be conducted in accordance with the ethical principles of the Declaration of Helsinki and GCP guidelines. Patient perspectives were considered during the design of this trial, including the selection of DCT elements intended to reduce participant burden. The original protocol (version 1.0) was approved by the Saga University Hospital IRB on 03 March 2025 (Approval No. Sadai-2024-12-03). The current amended protocol (version 1.4) was approved on 05 January 2026. The full study protocol is provided in this article and its supplementary materials. The statistical analysis plan will be available from the corresponding author upon reasonable request. The trial was prospectively registered with the Japan Registry of Clinical Trials (jRCT) (jRCT2071250003; https://jrct.mhlw.go.jp/latest-detail/jRCT2071250003) on 28 April 2025. Participant recruitment will be conducted from 1 August 2025–31 March 2027. Written informed consent will be obtained from each participant before enrollment. Consent from legally authorized representatives will not be accepted in this trial. When a participant is unable to read the informed consent documents because of impaired visual function, the informed consent process will be conducted in the presence of an impartial witness. If a participant experiences health-related harm associated with participation in this trial, appropriate medical care and other necessary treatment will be provided by the participating institution. In cases where the harm is determined to be related to this trial, compensation, including medical expenses and related allowances, will be provided in accordance with the terms and conditions of the clinical trial insurance policy and institutional regulations.

At the time of manuscript submission, participant recruitment is ongoing. Data collection is anticipated to be completed by 30 June 2027, and the study results are expected in late 2027. All data will be collected and handled confidentially by the investigators. Any important protocol modifications will be submitted to the IRB for approval prior to implementation and will be communicated to investigators, trial and partner sites, and relevant regulatory authorities as required. The results of this trial will be disseminated through publication in peer-reviewed journals and presentation at national and international conferences.

## Discussion

This trial is designed to generate prospective evidence on the clinical benefit and safety of subretinal tPA administration for SMH associated with AMD or RAM.

In Europe, a randomized prospective trial is underway to evaluate anti-VEGF therapy and surgical interventions, including vitrectomy, subretinal tPA administration, and intravitreal gas injection, for SMH associated with exudative AMD [41]. However, alteplase (Actilyse®), which is used in that trial, is not approved for manufacture and sale in Japan. To our knowledge, no studies have directly compared the efficacy or safety of different tPA preparations used for SMH. Although alteplase products other than Actilyse® are approved in Japan, excessive dilution may cause clouding; therefore, it is recommended that it be used at a concentration of at least 240,000 IU/mL. If the planned 8,000-IU dose were administered at 240,000 IU/mL, the injection volume would be approximately 33 µL, which may compromise dosing accuracy. By contrast, monteplase can be administered at a concentration consistent with the approved dilution conditions and was therefore selected for this trial.

The natural history of SMH varies according to hematoma volume and location. Small hematomas or hemorrhages that do not involve the fovea may regress spontaneously and have minimal visual impact. This trial targets patients with severe disease characterized by large hematomas unlikely to resolve spontaneously, foveal involvement, and anticipated severe visual impairment. The study uses a pre-post design to compare objective measures of hematoma burden and visual function before and after subretinal injection of monteplase. In this patient population, no treatment, gas tamponade with or without vitrectomy, or silicone oil tamponade with vitrectomy is unlikely to provide meaningful benefit, making inclusion of a control group ethically difficult to justify. In addition, subretinal administration of a placebo may cause complications such as retinal detachment, which further weakens the justification for a placebo group. Accordingly, this study was designed as a single-arm, open-label trial without a control group.

This trial targets patients with AMD or RAM, which are the main causes of SMH. Given the invasiveness and risks of subretinal injection, the study focuses on patients with moderate to severe disease. Accordingly, patients with CFT ≥ 300 µm and BCVA ≤0.2 will be included. Patients with organized thrombus considered difficult to dissolve, ophthalmic conditions that would interfere with efficacy or safety evaluation, or a high risk of bleeding will be excluded.

CFT is an objective OCT-derived parameter that reflects the combined thickness of the neurosensory retina and subfoveal hemorrhage. The treatment goal in SMH is to displace hemorrhage away from at least the fovea, and subfoveal hemorrhage thickness affects visual prognosis [2,8]. Intravitreal tPA combined with air exchange has also been reported to flatten hemorrhagic PED, and PED flattening may influence the need for additional treatment and visual prognosis [24]. Given the exploratory nature of this study, CFT was selected as the primary outcome because it can objectively capture subfoveal hemorrhage resolution and PED flattening, whereas change from baseline was used as a secondary outcome measure.

Retinal toxicity has been reported in patients who received two consecutive intravitreal injections of tPA 50 µg or a single intravitreal dose of 100 µg for SMH [42]. In Japan, previously reported subretinal doses of monteplase for SMH associated with AMD or RAM range from 4,000–32,000 IU (32–256 µg) [22,23,27,29]. At the main study site, Saga University Hospital, approximately 10 patients per year have received off-label tPA at doses of 5,000–10,000 IU (40–80 µg) by intravitreal or subretinal injection without major safety concerns. Accordingly, the trial dose was set at 8,000 IU (0.1 mL; tPA 64 µg), a dose supported by prior clinical experience for both safety and efficacy.

Possible AEs associated with monteplase administration include retinal toxicity, increased SMH, macular hole, vitreous hemorrhage, hyphema, endophthalmitis, elevated intraocular pressure, retinal detachment, corneal opacity, and cataract progression. These events may reflect not only the effects of the drug itself but also complications related to vitreous surgery and intraocular tamponade. To minimize these risks, participating institutions will be limited to centers with prior experience in subretinal tPA administration, and high-risk patients will be excluded according to the predefined exclusion criteria. Given the measures taken to minimize risk, the anticipated benefits of trial participation were considered to justify the potential risks associated with the intervention and study procedures.

Implementation of DCT requires operational systems such as eConsent, remote consultation platforms, and mechanisms for delegating selected trial activities to partner sites near participants [30]. However, implementation remains challenging because of regulatory heterogeneity, data-quality concerns, and digital inequality [33]. In addition, although the NIH policy mandating single-IRB review for multisite research aims to reduce duplication and improve efficiency, challenges remain, including interinstitutional governance, incorporation of local context, and communication with researchers [41]. To address these challenges, we established a DCT implementation framework and a single-IRB review system through a Japanese Agency for Medical Research and Development (AMED) project in 2023 (JP23yk0126023). In addition to developing institutional regulations at participating institutions, we conducted a simulated multicenter investigator-initiated clinical trial using a DCT approach together with a mock single-IRB review. As a result, approximately 1 year was required to develop the protocol, obtain agreement on the trial plan with the Pharmaceuticals and Medical Devices Agency (PMDA), establish the implementation framework, and secure IRB approval through the AMED project in 2024 (JP24yk0126029). Although we aimed to implement a single IRB, one participating institution conducted an independent ethical review. That institution is currently revising its internal regulations and establishing a system to enable future adoption of a single IRB.

Regarding delegation of trial-related duties to partner sites in Japan, a previous oncology report described a clinical trial in which contracts were executed between the lead trial site and neighboring hospitals already attended by the patients [43]. In the SACLA trial, it is difficult to predict when and where patients with SMH will develop symptoms, and the interval between enrollment and administration of the investigational product is short. Therefore, in addition to the conventional approach of enrolling participants after contract execution with partner sites, we developed a system that allows contract execution after enrollment through dynamic partnering (Fig 2). Compared with standard clinical trial contracts, contracts established through dynamic partnering require less preparation time, highlighting the importance of ongoing awareness and engagement activities related to DCTs. Dynamic partnering may facilitate enrollment from a broader geographic area and improve case accrual in acute, time-sensitive conditions.

This phase II study is designed to explore the efficacy of subretinal tPA administration. The intervention is intended primarily to displace hematomas rather than to treat the underlying diseases, such as AMD or RAM. Accordingly, long-term outcomes may be influenced by factors other than subretinal tPA administration. CFT, which is a surrogate marker of visual prognosis, and BCVA will be assessed for up to 12 weeks, whereas the presence or absence of a foveal hematoma measuring ≥1 disc diameter will be assessed for up to 4 weeks as an indicator of hematoma displacement. A long-term observational study with follow-up ophthalmic examinations is planned after completion of this trial.

Subretinal injection of monteplase may promote hematoma displacement and absorption and may improve visual prognosis. Patients who are currently unable to receive off-label subretinal injection of monteplase because of geographic or other barriers may gain access to this treatment through a DCT framework. In addition, adoption of a single IRB and dynamic partnering may allow this trial to be conducted more efficiently while supporting a more patient-centered model of clinical research. If successful, this model may offer a scalable approach for investigator-initiated ophthalmic trials targeting acute conditions that require rapid intervention. More broadly, because this model incorporates initiatives led by academic research organizations, it may have relevance beyond ophthalmology across other diseases and medical specialties.

## Supporting information

S1 Checklist SPIRIT checklist. (DOCX)

S2 Protocol Ethics-approved clinical trial protocol. (PDF)
