# Supplementary material for: Protocol for the SACLA trial: Efficacy and safety of subretinal monteplase for submacular hemorrhage in a phase II single-arm multicenter decentralized clinical trial
Source: PLoS One. 2026 Jul 2;21(7):e0353127. doi: 10.1371/journal.pone.0353127 (PMC13327112; doi:10.1371/journal.pone.0353127)
Supplement: S2 Protocol — (PDF) [file pone.0353127.s002.pdf]

Title of the Clinical Trial Protocol:

Efficacy and Safety of Subretinal Administration of Tissue Plasminogen Activator (Monteplase) for the Prevention of Vision Loss in Submacular Hemorrhage:

a Phase II Single-Arm Pre-Post Comparison Multicenter Investigator-Initiated Exploratory Clinical Trial

SACLA trial

Clinical Trial Protocol Number: [SCTR-O-01]

Version: 1.4

Investigational Product: Monteplase (recombinant)

Plain-Language Title: A Study of the Safety and Efficacy of a Tissue Plasminogen Activator Formulation for Submacular Hemorrhage

Study Acronym: SACLA trial

Study Phase: Phase II

jRCT Identifier: jRCT2071250003

Coordinating Investigator:

Clinical Research Center, University Hospital of Saga University

Specially Appointed Professor / Center Director

Noriko Yoshida

Date of Preparation: 17 December 2025

Contact information for the clinical trial coordinating physician and coordinating office: see Appendix 1.

Document History	
Document Version	Date
1.0	20 January 2025
1.1	24 April 2025
1.2	28 July 2025
1.3	26 September 2025
1.4	17 December 2025

Table of Contents

1. Summary of the Study Plan 8
1.1. Overview of the Clinical Trial Protocol 8
1.2. Study Flow Diagram 10
1.3. Schedule of Activities (SoA) 11
2. General Statements 12
2.1. Purpose of the Study 12
2.2. Background 12
2.3. Benefit–Risk Assessment 13
2.3.1. Risk Assessment 14
2.3.2. Benefit Assessment 14
2.3.3. Conclusion on Benefit–Risk Balance 14
3. Study Objectives, Endpoints, and Estimand 14
4. Study Design 19
4.1. Overview of Study Design 19
4.2. Rationale for Study Design 19
4.2.1. Subject Input on Study Design 20
4.3. Rationale for the Dose 20
4.4. Definition of Study Completion 20
5. Study Population 20
5.1. Inclusion Criteria 20
5.2. Exclusion Criteria 21
5.3. Considerations Regarding Lifestyle 22
5.3.1. Diet and Dietary Restrictions 22
5.3.2. Caffeine, Alcohol, Tobacco, and Other Habits 22
5.3.3. Physical Activity 22
5.3.4. Other Activities 22
5.4. Screening Dropouts 22
5.5. Criteria for Temporary Deferral of Enrollment 22
6. Study Intervention and Concomitant Therapies 22
6.1. Procedures for Study Intervention 22
6.1.1. Rescue Treatment 24
6.2. Preparation, Handling, Storage, and Management 24
6.3. Allocation of Trial Intervention 24
6.4. Blinding 25

6.5. Compliance with Trial Intervention 25
6.6. Dose Adjustment of Trial Intervention 25
6.7. Continuation of Trial Intervention After Trial Completion 25
6.8. Treatment of Overdose........ 26
6.9. Prior Therapy and Concomitant Therapy........ 26
6.9.1. Prior Therapy........ 26
6.9.2. Concomitant Medication / Concomitant Therapy........ 26
7. Discontinuation of Trial Intervention and Subject Discontinuation/Withdrawal........ 27
7.1. Discontinuation of Trial Intervention........ 27
7.2. Subject Discontinuation/Withdrawal........ 27
7.3. Lost to Follow-up........ 27
8. Trial Assessments and Procedures........ 28
8.1. Screening (Baseline) Assessment Procedures........ 28
8.1.1. Subject Background........ 28
8.1.2. Height and Weight........ 28
8.1.3. Chest X-ray........ 29
8.1.4. Electrocardiogram (ECG)........ 29
8.2. Efficacy Assessment Procedures........ 29
8.2.1. Optical Coherence Tomography (OCT) (both eyes)........ 29
8.2.2. Fundus Photography (both eyes)........ 29
8.2.3. Visual Acuity Testing (both eyes)........ 29
8.2.4. Intraocular Pressure Measurement (Visits 2/3/4: treated eye only; other visits: both eyes)........ 30
8.2.5. Anterior Segment Examination (Visits 2/3/4: treated eye only; other visits: both eyes)........ 30
8.2.6. Fundus Examination (Visits 2/4: treated eye only; other visits: both eyes)........ 31
8.2.7. Concomitant Medication / Concomitant Therapies........ 32
8.3. Safety Assessment Procedures........ 32
8.3.1. Vital Signs........ 32
8.3.2. Laboratory Blood Tests........ 32
8.3.3. Urinalysis........ 32
8.3.4. Clinical Laboratory Evaluations........ 32
8.3.5. Pregnancy Testing........ 33
8.4. Adverse Events, Serious Adverse Events, and Other Safety Reporting........ 33
8.4.1. Collection Period and Frequency for Adverse Events and Serious Adverse Events… 33
8.4.2. Identification of Adverse Events and Serious Adverse Events........ 33

8.4.3. Follow-up of Adverse Events and Serious Adverse Events 34
8.4.4. Regulatory Reporting Requirements for Serious Adverse Events 34
8.4.5. Pregnancy Information 34
8.4.6. Cardiovascular Events and Deaths 34
8.4.7. Disease-Related Events 35
8.5. Pharmacokinetics (PK) 35
8.6. Pharmacodynamics 35
8.7. Genetics 35
8.8. Biomarkers 35
8.9. Immunogenicity Assessment 35
8.10. Utilization of Medical Resources and Health Economics 35
9. Statistical Considerations 35
9.1. General Considerations 36
9.1.1. Statistical Hypothesis 36
9.1.2. Adjustment for Multiplicity 36
9.1.3. Impact of Strategy for Intercurrent Events 36
9.1.4. Handling of Missing Data 37
9.2. Analysis Populations 37
9.3. Analyses Supporting the Primary Objective 37
9.3.1. Primary Endpoint 37
9.3.2. Primary Analysis Methods 38
9.4. Analyses Supporting Secondary Objectives 38
9.4.1. Analyses Supporting Secondary Objectives 38
9.5. Exploratory Objective Analyses 39
9.6. Safety Analyses 39
9.7. Other Analyses 39
9.7.1. Other Variables and/or Parameters 39
9.7.2. Subgroup Analyses 40
9.8. Interim Analysis 40
9.9. Determination of Sample Size 40
10. Appendices 41
Appendix 1: 41
10.1. General Considerations Regarding Regulation, Ethics, and Trial Management 41
10.1.1. Regulatory and Ethical Considerations 41
10.1.2. Compliance with the Trial Protocol, Deviations, and Amendments 41
10.1.3. Amendments to the Clinical Trial Protocol 41

10.1.4. Institutional Review Board 42
10.1.5. Procedures for Explanation and Obtaining Informed Consent 42
10.1.6. Compensation for Health Injuries 43
10.1.7. Strategies for Subject Recruitment 43
10.1.8. Matters Concerning Protection of Subjects' Personal Information 44
10.1.9. Establishment of Committees 44
10.1.10. Data Quality Assurance 44
10.1.11. Identification of Source Data 45
10.1.12. Costs and Conflicts of Interest 45
10.1.13. Initiation and Termination of the Trial at the Trial Sites 46
10.1.14. Policy Regarding Disclosure 46
10.1.15. Secondary Use of Data 47
Appendix 2 48
10.2. Definitions, Severity, and Causality of Adverse Events and Serious Adverse Events ... 48
10.2.1. Detailed Definition and Description of Adverse Events 48
10.2.2. Definition of Serious Adverse Events 49
10.2.3. Recording and follow-up of Adverse Events and Serious Adverse Events 49
10.2.4. Reporting of Serious Adverse Events 51
10.2.5. Collection of Safety Information 52
Appendix 3 53
10.3. Guidance on Contraception 53
10.3.1. Definition of Women of Child-bearing Potential 53
10.3.2. Contraception Guidance 53
Appendix 4 54
10.4. Implementation at Partner Sites 54
Appendix 5 55
10.5. Preservation of Records 55
10.5.1. Trial Sites 55
10.5.2. Institutional Review Board 55
10.5.3. Sponsor-investigator and Trial Coordinating Physician 55
10.5.4. Preservation of Records at Other Institutions 55
Appendix 6 57
10.6. Revision History of the Clinical Trial Protocol 57
11. References 58

List of Abbreviations and Definitions

Abbreviation	English
AE	adverse event
AMD	age-related macular degeneration
CONSORT	consolidated standards of reporting trials
FSH	follicle stimulating hormone
GCP	good clinical practice
HRT	hormone replacement therapy
OCT	optical coherence tomography
PED	pigment epithelial detachment
PT-INR	prothrombin time-international normalized ratio
RAM	retinal arterial microaneurysm
RPE	retinal pigment epithelium
SAE	serious adverse event
SMH	submacular hemorrhage
tPA	tissue plasminogen activator
VEGF	vascular endothelial growth factor

1. Summary of the Study Plan

1.1. Overview of the Clinical Trial Protocol

◆ Protocol Title

A Multicenter, Single-Arm, Pre-Post Comparison, Phase II, Investigator-Initiated Clinical Trial to Evaluate the Efficacy and Safety of Sub-Retinal Administration of a Tissue Plasminogen Activator (Monteplase) Preparation for Sub-Macular Hemorrhage

◆ Short Title

A Study of the Safety and Efficacy of a Tissue Plasminogen Activator Preparation for Sub-Macular Hemorrhage

◆ Regulatory Agency Identifier

jRCT2071250003

◆ Rationale

Tissue plasminogen activator (tPA) is an enzyme involved in the fibrinolytic system; it forms a complex with fibrin and activates plasminogen to plasmin, thereby degrading fibrin and exerting thrombolytic activity. In patients with sub-macular hemorrhage (SMH), sub-retinal administration of tPA has been reported (off-label use) to reduce fibrin-mediated photoreceptor and retinal pigment epithelium injury, thereby mitigating visual loss. This Phase II, investigator-initiated trial is planned to assess the efficacy and safety of sub-retinal tPA (Monteplase, recombinant) administered in conjunction with vitrectomy, with the aim of expanding the indication of tPA or developing an ophthalmic formulation for the severe ophthalmic condition of SMH.

◆ Objectives and Endpoints

Objective	Endpoints
Primary	
To evaluate the efficacy of sub-retinal Monteplase (recombinant) on central macular hemorrhage in patients with SMH secondary to AMD or RAM.	Change in central foveal thickness(CFT) from baseline at Week 1.
Secondary (Efficacy)	
Evaluation of the efficacy of sub-retinal Monteplase (recombinant) in patients with	<Efficacy Evaluation Items><br>1) Change in CFT from baseline at Week 4.

SMH secondary to AMD or RAM, using ophthalmic examination findings as indices.	2) Presence or absence of a foveal hemorrhage measuring ≥1 disc diameter at Week 4.<br>3) BCVA (logMAR equivalent) at Week 4.<br>4) Change in BCVA (logMAR equivalent) from baseline at Week 4.<br>5) Change in CFT from baseline at Week 12.<br>6) BCVA (logMAR equivalent) at Week 12.<br>7) Change in BCVA (logMAR equivalent) from baseline at Week 12.
Secondary (safety)	
To evaluate the safety of subretinal administration of monteplase (recombinant) in patients with SMH.	Adverse events

◆ Overview of the Overall Design

Intervention model: Single-arm

Control: Baseline control

Active comparator: None

Allocation method: None

Blinding: None

Target population type: Adult patients

Target population diagnosis: Submacular hemorrhage due to AMD or RAM

Target population age range: Minimum 18 years; no maximum

Distribution of study sites: Multicenter collaborative

Trial phase: Phase II

Committee: None

Duration per subject: 12-week observation period after enrollment

Planned sample size: 20 subjects

◆ Brief Summary of Trial Intervention and Observation

After obtaining written informed consent from the patient, those who satisfy all inclusion criteria and do not meet any exclusion criteria will be enrolled. Enrolled subjects will undergo pars plana vitrectomy, followed by subretinal administration of the investigational product diluted to 80,000 IU/mL, 0.1 mL (8,000 IU). Subsequently, a gas tamponade or silicone-oil exchange will be performed (Day 1). The primary endpoint will be assessed at 1 week post-

procedure (Day 8, Week 1), and secondary efficacy endpoints will be observed at 1 month (Day 29, Week 4) and 3 months (Day 85, Week 12) post-procedure. Adverse events will be collected throughout the observation period for safety evaluation.

◆ Trial Implementation Period

1 August 2025 – 31 October 2027 (Enrollment period: through 31 March 2027; Observation period: through 30 June 2027)

1.2. Study Flow Diagram

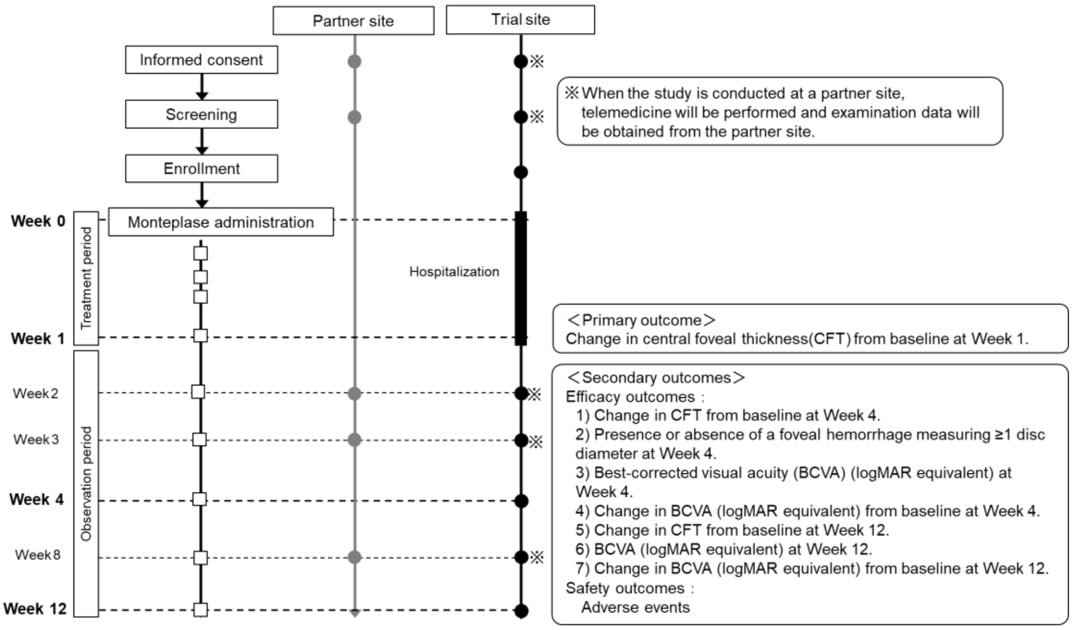

1.3. Schedule of Activities (SoA)

Activity	Consent	Screening*2	Enrollment	Treatment period					Observation period					Discontinuation*6
Visit		Within 7 days before registration		Visit 1	Visit 2	Visit 3	Visit 4	Visit 5	Visit 6	Visit 7	Visit 8	Visit 9	Visit 10	
Week				0				1	2	3	4	8	12	
Day				1	2	3	4	8	15	22	29	57	85	
Visit window (Day)				Within 7days after registraion	—	—	—	-1～+3	±3	±3	±3	±7	±7	+7
Intervention				○										
Trial site	Telemedicine	Telemedicine / In-person visit	Enrollment	Hospitalization					Telemedicine	Telemedicine	In-person visit	Telemedicine	In-person visit	Telemedicine / In-person visit
Partner site*1	In-person visit	In-person visit							In-person visit	In-person visit		In-person visit		In-person visit
Informed consent	○													
Registgration			○											
Medical and ophthalmic history		○												
Height・Weight		○												
Vital signs		○		○*4	○	○	○	○			○		○	○
Fundus camera・OCT examination		○*3						○	○	○	○	○	○	○
Visual acuity examination		○*3						○	○	○	○	○	○	○
Intraocular pressure・Slit-lamp examination		○*3			○*5	○*5	○*5	○	○	○	○	○	○	○
Fundus examination		○*3			○*5		○*5	○	○	○	○	○	○	○
Blood test		○*3				○								○*7
Urine test		○*3				○								○*7
Pregnancy test		○												
Chest X-ray		○*3												
Electrocardiogram		○*3												
Concomitant medications / therapies		←												→
Adverse events			←											→

*1 If a partner medical institution is not available, the clinical trial site shall be visited.

*2 Either the partner medical institution or the trial site may be used. When multiple test results are available within the period, the data closest to the enrollment date shall be used.

*3 If data are available within 7 days after enrollment, they may be used even before consent acquisition.
*4 Measured before and after dosing.
*5 Performed only on the treated eye.
*6 May be performed at the trial site or at the partner site. If performed at the partner site, the trial site will provide remote medical care.
*7 Performed only if discontinuation occurs before Visit 3.

2. General Statements

"Monteplase (recombinant)" is a glycoprotein produced from a modified human tPA-derived gene in which the cysteine at the 84th amino-acid residue of the N-terminal region of human tissue-type plasminogen activator (tPA) is replaced by serine; it dissolves thrombi by activating plasminogen in the clot to plasmin. In Japan, intravenous administration for dissolution of coronary-artery thrombi in acute myocardial infarction and pulmonary-artery thrombi in acute pulmonary embolism has already been approved; however, we are now attempting to develop an ophthalmic formulation intended to dissolve thrombi in submacular hemorrhage (SMH).

2.1. Purpose of the Study

To evaluate the efficacy and safety of sub-retinal administration of Monteplase (recombinant) for submacular hemorrhage that causes severe visual-function impairment.

2.2. Background

SMH is characterized by accumulation of blood beneath the retina, arising from progression of age-related macular degeneration (AMD) or rupture of retinal arterial microaneurysms (RAM). SMH induces retinal damage primarily through toxic, tractional, and barrier effects[1]. The toxic effect is attributable to several substances; a representative example is iron toxicity from erythrocyte-derived ferritin, which damages the choroidal-retinal circulation, photoreceptors, and the retinal pigment epithelium (RPE). The tractional effect results from fibrin generated by the SMH, causing photoreceptor traction and degeneration. The barrier effect interferes with the movement of nutrients and metabolites between the choroid and the outer retinal layers due to the physical presence of SMH. SMH causes acute and severe visual impairment, and in the natural course, over 80 % of patients end with a final visual acuity of less than 0.1[2]. The incidence of SMH with optic-disc diameter ≥2 disc diameters is reported as 5.4−25 cases per 1,000,000 persons per year, meeting the definition of a rare disease[3, 4].

Seventy to ninety percent of SMH cases are caused by AMD, and 5–30 % by RAM[5]. In a 10-year observational study of patients with exudative AMD receiving vascular endothelial growth

factor (VEGF) inhibitors, the annual incidence of SMH associated with visual loss was estimated at 0.46 %[6]. AMD is a leading cause of blindness in developed countries, and in Japan, macular degeneration including AMD ranks fourth among causes of visual disability[7]. The prevalence of AMD is estimated at 8.7 % of the world population; because aging is the primary risk factor, a substantial increase in patients is projected worldwide, especially in Asia, during the era of global aging[8]. In a large cohort study in Japan covering the entire population aged ≥40 years (approximately 76 million), about 246 000 active exudative AMD patients were identified between 2011 and 2018, and prevalence has steadily increased across almost all age groups ≥65 years[9]. In Japan, the proportion of exudative AMD patients is higher than in Europe and the United States; consequently, the number of SMH cases are expected to rise with the increasing patient population. RAM occurs in the elderly, is more common in women, and is strongly associated with hypertension and arteriosclerosis[10]. The visual prognosis of RAM depends on macular pathology and may be complicated by intra-retinal hemorrhage or secondary macular holes; consequently, eyes with SMH in Japan are generally reported to have poor visual outcomes[11].

When SMH becomes chronic, it induces irreversible neuro-retinal degeneration that adversely affects visual prognosis[12-14], therefore, the therapeutic goal for SMH is to remove the hemorrhage from the macular region, or at least from the fovea, as promptly as possible[5]. The clinical characteristics of SMH—including cause, location, volume, and duration—are heterogeneous, and a definitive treatment guideline has not yet been established. Current management is selected according to the underlying disease and the state of the hemorrhage and includes administration of VEGF-inhibitors aimed at reducing leakage from choroidal neovascularization, intravitreal injection of expansile gas to facilitate hemorrhage displacement, sub-retinal hemorrhage evacuation, retinal photocoagulation, and other disease-specific interventions. tPA is an enzyme involved in the fibrinolytic system; it forms a complex with fibrin and activates plasminogen to plasmin, thereby degrading fibrin and exerting thrombolytic activity. In the early phase of SMH, fibrin induces rupture of the photoreceptor inner and outer segments, and with time leads to destruction of the full retinal thickness. However, Lim et al. reported in 1995 that SMH containing tPA did not form fibrin strands and did not cause photoreceptor rupture[15]. In 1996, Herriot et al. reported treatment of SMH with tPA combined with gas injection. Intravitreal or sub-retinal administration of tPA is thought to reduce fibrin-mediated damage to photoreceptors and the retinal pigment epithelium; when combined with vitrectomy or intravitreal gas, it may promote removal of the lysed clot and thereby mitigate visual loss. Ocular local tPA therapy may be performed with or without concomitant VEGF-inhibitor, via intravitreal injection, or as sub-retinal injection in conjunction with vitrectomy. A review of multiple studies summarizing visual-acuity changes before and after

ocular tPA therapy indicated that the greatest visual improvement was observed with the combination of vitrectomy, sub-retinal tPA injection, intravitreal gas injection, and VEGF-inhibitor therapy[17].

As of January 2025, tPA is being used off-label worldwide for SMH. In Europe, a randomized prospective trial (the TIGER study: ″Vitrectomy, subretinal Tissue plasminogen activator and Intravitreal Gas for submacular hemorrhage secondary to Exudative Age-Related macular degeneration″, a phase 3, pan-European, two-group, non-commercial, active-control, observer-masked, superiority, randomized controlled surgical trial) is underway to evaluate VEGF-inhibitor monotherapy versus surgical therapy (vitrectomy, sub-retinal tPA injection, intravitreal gas injection, and VEGF-inhibitor administration)[18]. The alteplase (Actilyse®) used in that trial has not received marketing authorization in Japan.

In Japan, several reports have described the efficacy and safety of ocular local tPA therapy for SMH secondary to AMD or RAM[5, 19-31]. Nevertheless, tPA remains unapproved for SMH, and off-label use continues in clinical practice. Off-label use is governed by the "Standards for the Use of Unapproved New Drugs, etc., as Determined by the Minister of Health, Labour and Welfare under Article 9-23, Paragraph 1, Item 8-ro of the Medical Care Act Enforcement Ordinance" (Notification from the Director of the Medical Affairs Division, Ministry of Health, Labour and Welfare, 10 June 2016). Accordingly, each institution′s Unapproved New Drug Evaluation Committee conducts an ethical review and determines the appropriateness of use. When use is approved, all treated cases must be reported regularly. Consequently, only a limited number of facilities are able to administer tPA, and no alternative hemolytic agent to tPA is available for displacing sub-macular hemorrhage. Physical constraints—such as difficulty travelling to distant centers due to visual impairment—lead to treatment delays or abandonment for many patients. Therefore, the investigators aim to expand the indication for tPA in the severe ophthalmic condition of SMH, or to develop an ophthalmic formulation, by planning a phase II physician-initiated clinical trial to evaluate the efficacy and safety of sub-retinal tPA administration.

2.3. Benefit–Risk Assessment

More detailed information on the known and anticipated benefits and risks of Monteplase (recombinant) and on the reasonably foreseeable adverse events (AE) will be provided in the investigational product dossier for this clinical trial.

2.3.1. Risk Assessment

Adverse events that may occur with administration of monteplase (genetically engineered) include retinal toxicity (retinal morphological changes, etc.), increased sub-macular

hemorrhage, macular hole, vitreous hemorrhage, anterior chamber hemorrhage, endophthalmitis, elevated intra-ocular pressure, retinal detachment, corneal opacity, progression of cataract, etc. In addition to pharmacologic effects of the drug itself, events associated with vitreous surgery or intra-ocular tamponade are also encompassed.

To minimize these risks, the trial sites for this study shall be facilities that have prior experience with sub-retinal administration of tPA preparations, and patients at high risk will be excluded by the exclusion criteria.

2.3.2. Benefit Assessment

Subjects are expected to benefit from sub-retinal administration of monteplase (genetically engineered) through promotion of hematoma displacement and absorption, leading to improvement in visual prognosis. Furthermore, patients who currently cannot receive sub-retinal monteplase (genetically engineered) because of off-label use and geographic constraints may obtain treatment by participating in this decentralized clinical trial.

2.3.3. Conclusion on Benefit–Risk Balance

Considering the measures taken to minimize risk for subjects participating in this trial, the potential risks identified in relation to the investigational intervention and study procedures are justified by the anticipated benefits to subjects with sub-macular hemorrhage.

3. Study Objectives, Endpoints and Estimand

Objective	Endpoints
Primary	
To evaluate the efficacy of sub-retinal Monteplase (recombinant) on central macular hemorrhage in patients with SMH secondary to AMD or RAM.	Change in central foveal thickness(CFT) from baseline at Week 1.
Secondary (Efficacy)	
Evaluation of the efficacy of sub-retinal Monteplase (recombinant) in patients with SMH secondary to AMD or RAM, using ophthalmic examination findings as indices.	<Efficacy Evaluation Items><br>1) Change in CFT from baseline at Week 4.<br>2) Presence or absence of a foveal hemorrhage measuring ≥1 disc diameter at Week 4.<br>3) BCVA (logMAR equivalent) at Week 4.<br>4) Change in BCVA (logMAR equivalent) from baseline at Week 4.

	5) Change in CFT from baseline at Week 12.<br>6) BCVA (logMAR equivalent) at Week 12.<br>7) Change in BCVA (logMAR equivalent) from baseline at Week 12.
Secondary (safety)	
To evaluate the safety of subretinal administration of monteplase (recombinant) in patients with SMH.	Adverse events

<Estimand for the Primary Objective>

Primary clinical question: In patients with SMH due to AMD or RAM, regardless of the use of VEGF-inhibitor therapy or the use of tPA formulations for other diseases, what is the change in central foveal hemorrhage volume at the 1-week time point after subretinal administration of monteplase (recombinant)?

i. Treatment condition of interest	Subretinal administration of monteplase (recombinant)
ii. Population	Patients with SMH defined by the inclusion criteria (severe sub-macular hemorrhage in which spontaneous resolution is not expected, the bleed involves the fovea, and marked visual-acuity loss is anticipated)
iii. Endpoint	Change from baseline to week 1 in CFT measured by optical coherence tomography (OCT)
iv. Other intercurrent events	See Table 3-1
v. Population-level summary	Mean change from baseline to week 1 in CFT

Rationale for the estimand: In SMH, the thickness of the central foveal hemorrhage is known to affect visual prognosis. CFT measured by OCT represents the combined thickness of the retina and the hemorrhage at the fovea, thereby reflecting the volume of the central foveal hemorrhage and also allowing assessment of hemorrhagic pigment-epithelial detachment, which is related to visual outcome. Consequently, CFT measured by OCT was selected as the variable constituting the primary estimand. Even when considering the potential impact of VEGF-inhibitor use after monteplase administration or the use of tPA formulations for other diseases, an early reduction in OCT-measured CFT is considered important for regulatory authorities and for individuals involved in determining the therapeutic strategy for the target patient population. Moreover, the persistence of SMH adversely affects visual prognosis; therefore, early displacement of the SMH is important. Accordingly, the 1-week time point, at

which OCT measurement of CFT can be performed without interference from gas tamponade introduced during vitrectomy, was designated as the assessment time point for the primary estimand.

<Estimand for the Secondary Objective (1)>

Clinical question of interest: In patients with SMH due to AMD or RAM, regardless of the use of VEGF-inhibitor therapy or the use of tPA formulations for other diseases, what is the change in ophthalmic examination findings at the 4-week time point after subretinal administration of monteplase (recombinant)?

i. Treatment condition of interest	Subretinal administration of monteplase (recombinant)
ii. Population	Patients with SMH defined by the inclusion criteria (severe sub-macular hemorrhage in which spontaneous resolution is not expected, the bleed involves the fovea, and marked visual-acuity loss is anticipated)
iii. Endpoint	1) Change in CFT from baseline at Week 4.<br>2) Presence or absence of a foveal hemorrhage measuring ≥1 disc diameter at Week 4.<br>3) BCVA (logMAR equivalent) at Week 4.<br>4) Change in BCVA (logMAR equivalent) from baseline at Week 4.
iv. Other intercurrent events	See Table 3-1
v. Population-level summary	1) Mean change from baseline to Week 4 in CFT measured by OCT.<br>2) Proportion of patients with foveal hemorrhage measuring ≥1 disc diameter observed by fundus photography at Week 4.<br>3) Mean BCVA (logMAR equivalent) at Week 4.<br>4) Mean change from baseline to Week 4 in BCVA (logMAR equivalent).

Rationale for the estimand: SMH causes acute and severe visual impairment, and prolonged hemorrhage may induce irreversible degeneration of the neurosensory retina, thereby affecting visual prognosis. Even when considering the effects of VEGF inhibitor use after monteplase administration or the use of tPA products for other diseases, reduction of the hematoma,

displacement of the hematoma, and the degree of visual recovery are considered important for regulatory authorities and individuals involved in treatment decision-making for the target patient population. Although long-term observation is required to evaluate visual recovery, hematomas may undergo spontaneous absorption over time, and rebleeding frequently occurs. Therefore, long-term changes in CFT may be substantially influenced by factors other than subretinal tPA administration. Accordingly, one month was selected as one of the assessment time points for the secondary estimand.

<Estimand for the Secondary Objective (2)>
Clinical question of interest: In patients with SMH secondary to AMD or RAM, regardless of the use of VEGF inhibitors or tPA products for other diseases, to what extent do ophthalmologic findings change at Week 12 following subretinal administration of monteplase (recombinant)?

i. Treatment condition of interest	Subretinal administration of monteplase (recombinant)
ii. Population	Patients with SMH defined by the inclusion criteria (severe sub-macular hemorrhage in which spontaneous resolution is not expected, the bleed involves the fovea, and marked visual-acuity loss is anticipated)
iii. Endpoint	1) Change in CFT from baseline at Week 12.<br>2) BCVA (logMAR equivalent) at Week 12.<br>3) Change in BCVA (logMAR equivalent) from baseline at Week 12.
iv. Other intercurrent events	See Table 3-1
v. Population-level summary	1) Mean change from baseline to Week 12 in CFT measured by OCT.<br>2) Mean BCVA (logMAR equivalent) at Week 12.<br>3) Mean change from baseline to Week 12 in BCVA (logMAR equivalent).

Rationale for the estimand: SMH causes an acute and severe visual impairment, and when it becomes chronic it induces irreversible neuro-retinal degeneration that affects visual prognosis. Even when the impact of VEGF-inhibitor uses after monteplase administration, or the use of tPA formulations for other diseases, is taken into account, the reduction of the hemorrhage, the presence or absence of hemorrhage displacement, and the degree of visual recovery are considered important to regulatory authorities and to those determining the therapeutic strategy for the target patient population. Although observation of visual recovery requires a long period,

the hemorrhage is naturally reabsorbed over time and many patients experience re-bleeding; therefore, long-term changes in CFT may be increasingly influenced by factors other than sub-retinal tPA administration. Consequently, the 3-month time point was selected as one of the assessment points for a secondary estimand.

Table 3-1 Other Intercurrent Events

Intermediate events		Reasons for occurrence	Primary estimand	Secondary estimand						
			Central foveal thickness			Best-corrected visual acuity (logMAR equivalent)				Presence of hemoprrhage measuring at least 1 optic-disc diameter
			Change from baseline to Week 1	Change from baseline to Week 4	Change from baseline to Week 12	Value at Week 4	Change from baseline to Week 4	Value at Week 12	Change from baseline to Week 12	Presence of hemoprrhage measuring at least 1 optic-disc diameter at Week 4
Vitreous re-operation		Re-bleeding§	Composite variable	Composite variable	Composite variable	Composite variable	Composite variable	Composite variable	Composite variable	Composite variable
		Ocular complications (endophthalmitis, retinal detachment, vitreous hemorrhage)	Treatment policy	Treatment policy	Treatment policy	Treatment policy	Treatment policy	Treatment policy	Treatment policy	Treatment policy
Sub-Tenon administration of triamcinolone acetonide		Macular edema	Treatment policy	Treatment policy	Treatment policy	Treatment policy	Treatment policy	Treatment policy	Treatment policy	Treatment policy
lwithdrawal of consebt / loss to follow-up		Withdrawal of consent for reasons that have a major impact on the evaluation items (e.g., serious adverse events)	Composite variable	Composite variable	Composite variable	Composite variable	Composite variable	Composite variable	Composite variable	Composite variable
		Withdrawal of consent or loss to follow-up for reasons other than those described above	Virtual	Virtual	Virtual	Virtual	Virtual	Virtual	Virtual	Virtual
Implementation of rescue therapy	Photodynamic therapy	Treatment of underlying disease	Treatment policy	Treatment policy	Treatment policy	Treatment policy	Treatment policy	Treatment policy	Treatment policy	Treatment policy
	VEGF inhibitor – Treatment	Treatment of underlying disease	Treatment policy	Treatment policy	Treatment policy	Treatment policy	Treatment policy	Treatment policy	Treatment policy	Treatment policy
	Intravitreal gas injection	Re-bleeding	Virtual	Virtual	Virtual	Virtual	Virtual	Virtual	Virtual	Virtual
mplementation of prohibited concomitant drugs / prohibited concomitant therapy	Defibrinid sodium	Hepatic central-vein occlusion	Treatment policy	Treatment policy	Treatment policy	Treatment policy	Treatment policy	Treatment policy	Treatment policy	Treatment policy
	Intravenous, sub-retinal, or intravitreal administration of tPA preparations other than monteplase (recombinant)	Additional treatment for SMH	Virtual	Virtual	Virtual	Virtual	Virtual	Virtual	Virtual	Virtual
		Treatment of other diseases (acute myocardial infarction, cerebral infarction)	Treatment policy	Treatment policy	Treatment policy	Treatment policy	Treatment policy	Treatment policy	Treatment policy	Treatment policy
	Intravenous or intravitreal administration of monteplase (recombinant)	Additional treatment for SMH	Virtual	Virtual	Virtual	Virtual	Virtual	Virtual	Virtual	Virtual
		Treatment of other diseases (acute myocardial infarction, acute pulmonary embolism)	Treatment policy	Treatment policy	Treatment policy	Treatment policy	Treatment policy	Treatment policy	Treatment policy	Treatment policy
	Hemorrhage removal surgery	Additional treatment for SMH	Composite variable	Composite variable	Composite variable	Composite variable	Composite variable	Composite variable	Composite variable	Composite variable

§Including cases in which a tPA formulation (including monteplase (recombinant)) is administered via subretinal injection.

4. Study Design

4.1. Overview of Study Design

Intervention Model: Single-group

Control: Baseline control

Active Comparator: None

Allocation Method: None

Masking: None

Target Population Type: Adult subjects

Target Population Diagnosis: SMH secondary to AMD or RAM

Target Population Age Range: Minimum 18 years, no upper limit

Distribution of Study Sites: Multicenter collaborative

Trial Phase: Phase II
Committee: None
Duration of Participation per Subject: 12-week observation period after enrollment

4.2. Rationale for Study Design

<Single-Group Trial>

The natural course of SMH varies according to hematoma volume and location of bleeding. Small hematomas or hemorrhages that do not involve the fovea may resolve spontaneously with minimal impact on visual acuity. In this study, we will enroll severe patients in whom a large hematoma is unlikely to resolve spontaneously and whose bleeding involves the fovea, resulting in a predicted marked loss of visual acuity. The study will therefore be a pre-post comparison trial evaluating objective measurements reflecting hematoma volume and visual function before and after tPA administration. In the patient population targeted by this trial, improvement of the disease state is not expected with no treatment, vitrectomy with gas tamponade, or vitrectomy with oil tamponade; consequently, establishing a control arm would be ethically unacceptable. Moreover, sub-retinal administration of a placebo could cause complications such as retinal detachment, rendering a placebo arm ethically unacceptable. Accordingly, the trial will be conducted without a control group as an open-label, single-arm study of the investigational product.

<Target Population>

Patients whose underlying disease is AMD or RAM, the principal causes of SMH, will be eligible. Considering the invasiveness and risk of sub-retinal administration, only patients with at least moderate severity will be enrolled: those with a CFT ≥ 300 μm and a BCVA ≤ 0.2 (decimal). Patients in whom the clot is organized and unlikely to dissolve, those with concomitant ocular diseases that could affect efficacy or safety assessments, and those at high risk of further bleeding will be excluded.

<Evaluation Items>

Central foveal thickness is an objective measurement obtained by OCT, representing the sum of retinal thickness and hematoma volume at the fovea. The therapeutic goal for SMH is to remove the hemorrhage, at minimum from the foveal region[5], and the thickness of sub-foveal hemorrhage influences visual prognosis[12]. In addition, flattening of hemorrhagic pigment epithelial detachment (PED) has been reported as an effect of intravitreal tPA injection combined with air replacement, and PED flattening may influence the number of additional treatments required and visual prognosis[23]. Considering that this study is exploratory in nature,

CFT, which enables objective evaluation of subfoveal hemorrhage resolution and PED flattening, was selected as the variable for the primary estimand, and the change from baseline was set as the primary endpoint.

4.2.1. Subject Input on Study Design

Not applicable

4.3. Rationale for the Dose

Retinal toxicity has been reported in patients who received intravitreal tPA 50 μg twice consecutively or 100 μg for submacular hemorrhage (SMH)[32]. In Japan, reported sub-retinal doses of monteplase for SMH secondary to AMD or RAM range from 4,000−32,000 IU (32 μg−256 μg)[19, 21, 25, 31]. At the principal site of this trial, Saga University Hospital, off-label use of tPA 5,000−10,000 IU (40 μg−80 μg) has been administered intravitreally or sub-retinally to approximately ten patients per year without safety concerns. Consequently, this trial will employ a dose of 8,000 IU (0.1 mL, tPA 64 μg), which has demonstrated consistent safety and efficacy, as the prescribed dose.

4.4. Definition of Study Completion

The termination of the study is defined as the date of the final assessment of the last subject enrolled in the trial.

Each subject will be considered to have completed the trial upon completion of the 12-week assessment.

5. Study Population

No deviations from the eligibility criteria as specified in the clinical trial protocol will be permitted.

5.1. Inclusion Criteria

Subjects are eligible to participate in this trial only if they meet all of the following criteria:

Age:

1) Patient aged ≥ 18 years at the time of signing the informed consent form.

Disease Characteristics:

2) Patient with sub-macular hemorrhage involving the fovea secondary to AMD or RAM in the study eye.

3) At screening, CFT measured by OCT in the study eye is ≥ 300 μm.
4) At screening, BCVA in the study eye is ≤ 0.2 (decimal).

Other:
5) Patient who can provide written informed consent; if visual impairment makes document review difficult, verbal consent may be obtained from the subject together with the signature of a witness.
6) (For women of child-bearing potential) Patient who, from the time informed consent is obtained until 2 days after investigational product administration, agrees to use an appropriate method of contraception*.
* See 10.3.2.

5.2. Exclusion Criteria
If a subject meets any one of the following criteria, the subject shall be excluded from the trial:

Condition:
1) Patients who, at the time of screening, are judged by the principal investigator or sub-investigator to have had a sub-macular hemorrhage (SMH) for a prolonged period with evident organization or fibrosis.
2) Patients who, at the time of screening, have active proliferative diabetic retinopathy, uveitis, or optic neuritis.
3) Patients who, in the judgment of the principal investigator or sub-investigator, have ocular comorbidities such as macular atrophy or fibrosis that make visual improvement difficult.

Matters that may affect trial intervention or assessment:
4) Patients who, at the time of screening, have anterior-segment or vitreous abnormalities that could interfere with fundus observation by OCT, color fundus photography, or fluorescein angiography.
5) Patients with a known allergy to, or a history of allergy to, any component of the investigational product.
6) Patients with alcohol dependence, drug dependence, or psychiatric disorders that would interfere with participation in the trial.
7) Patients who are scheduled to undergo ophthalmic surgery other than administration of the investigational product during the trial period.

Matters that may affect safety:

8) Patients with active bleeding (e.g., gastrointestinal bleeding, urinary-tract bleeding, retroperitoneal bleeding, intracranial hemorrhage, hemoptysis).
9) Patients who have undergone intracranial or spinal surgery or have sustained an intracranial or spinal injury within two months prior to obtaining informed consent.
10) Patients with intracranial tumors, arteriovenous malformations, or aneurysms.
11) Patients younger than 70 years of age with a prothrombin time−international normalized ratio (PT-INR) ≥ 3.1, and patients aged ≥ 70 years with PT-INR ≥ 2.7 (unless it is anticipated that the INR can be reduced below these levels before vitreous surgery, taking into account the balance between systemic risk and intra-ocular hemorrhage risk; in such cases, PT-INR < 2.7 must be confirmed by laboratory testing on the day of surgery).
12) Patients with hypertension whose systolic blood pressure remains ≥ 180 mmHg despite antihypertensive therapy.
13) Women of child-bearing potential who are not using adequate contraception, pregnant women, women who may be pregnant, lactating women, or subjects who plan to become pregnant during the trial period.

Other:
14) Patients currently participating in another clinical trial, or who have participated in another trial within six months prior to obtaining informed consent.
15) Patients deemed unsuitable by the principal investigator or sub-investigator.

5.3. Considerations Regarding Lifestyle

5.3.1. Diet and Dietary Restrictions
No special restrictions.

5.3.2. Caffeine, Alcohol, Tobacco, and Other Habits
No special restrictions.

5.3.3. Physical Activity
If gas tamponade has been performed, the subject must not travel by air until the intra-ocular gas bubble has disappeared (because the gas bubble may expand and cause an increase in intra-ocular pressure). The subject should also avoid going to high altitudes or mountainous areas. Caution is also required with rapid ascent in elevators.

5.3.4. Other Activities

If gas tamponade has been performed, the subject must not receive hyperbaric oxygen therapy until the intra-ocular gas bubble has disappeared.

5.4. Screening Dropouts

Screening dropout occurs when a subject who has consented to participate in the clinical trial subsequently fails to meet any of the inclusion criteria or meets any of the exclusion criteria. To satisfy CONSORT reporting requirements and to respond to inquiries from regulatory authorities, minimal information (demographic characteristics and the reason for screening dropout) shall be recorded for subjects who are deemed ineligible at screening.
When medically and ethically appropriate, rescreening may be permitted for subjects who experienced screening dropout. If rescreening is performed, informed consent shall be obtained again and a new subject identification number shall be assigned.

5.5. Criteria for Temporary Deferral of Enrollment

Not applicable.

6. Study Interventions and Concomitant Therapies

Study interventions refer to all pre-specified investigational products, non-investigational study drugs, medical devices, and other interventions (e.g., surgical or behavioral interventions) that are used or performed on subjects during the conduct of the trial.

6.1. Procedures for Study Interventions

The study interventions to be administered are listed in Table 6-1, and the procedures for the study interventions are described in Table 6-2.

Table 6-1. Interventions to Be Performed

Intervention Label	Monteplase (recombinant)	Vitrectomy	Gas tamponade※1	Silicone oil※1
Intervention Name	Japanese name: Monteplase (recombinant) (JAN)<br>English name: Monteplase (genetical recombination) (JAN)	Vitrectomy	Air, sulfur hexafluoride (SF_6) or octafluoropropane (C_3F_8)	Silicone-oil replacement
Dosage Form /	Injectable solution (vial)	—	All are	colorless

Physical Characteristics	clear, colorless liquid		colorless and odorless gases	liquid
Specification	One vial (5 mL) containing 400,000 International Units (IU)	—	—	—
Dosage and Administration	Add 5 mL of physiological saline to one vial of monteplase (recombinant), dissolve completely without foaming to obtain a solution of 80,000 IU/mL; administer 0.1 mL (8,000 IU) sub-retinally after vitrectomy. If injection is difficult, the surgeon may, at his/her discretion, reduce the dose as appropriate.	—	Intra-ocular injection	Intra-ocular injection
Administration Frequency	Once	Once	Once	Once
Route of Administration	Sub-retinal administration	—	Intravitreal administrati on	Intravitreal administrati on
Purpose	Investigational product	Adjunctive surgery	Tamponade	Tamponade
Packaging and Labeling	Provided with the following information:<br>1) Indication that it is for clinical-trial use<br>2) Name and address of the clinical-trial coordinating physician<br>3) Chemical name or identifier<br>4) Batch number or	—	—	—

	manufacturing code<br>5) Storage conditions and expiration date			
Storage	Store at room temperature (1 – 30 °C) (room temperature according to the Japanese Pharmacopoeia)	—		

※1 Select one.

Table 6-2

Study-Arm Title	Active Drug Group
Intervention procedure	1) Perform local anesthesia or general anesthesia.<br>2) Conduct pars plana vitrectomy using a 3-port system with 23, 25 or 27-gauge instrumentation.<br>3) Phacoemulsification and intraocular lens implantation shall be performed at the discretion of the surgeon and according to the subject's preference.<br>4) Peeling of epiretinal membrane over the macula and internal limiting membrane is permitted.<br>5) Monteplase (recombinant) shall be administered with a sub-retinal injection needle into the sub-retinal hemorrhagic area located away from the fovea. Injection under the RPE and areas with RPE detachment shall be avoided to prevent RPE rupture. Multiple injection sites may be used as needed. During injection, intra-ocular pressure shall be monitored by palpation.<br>6) If retinal breaks are present, treat them with laser photocoagulation or cryotherapy.<br>7) Perform gas tamponade or silicone-oil exchange (the surgeon shall select either method based on the subject's severity, feasibility of positioning restrictions, etc.).<br>8) After monteplase (recombinant) administration, the subject shall remain in a supine or prone position for 20–60 minutes (including intra-operative time) according to the extent of hemorrhage. Thereafter, positioning restrictions shall be

	applied as directed by the surgeon, and the details shall be recorded in the case report form.
Related Intervention Labels	Monteplase (recombinant), vitrectomy, gas tamponade or silicone-oil exchange

6.1.1. Rescue Treatment

If the principal investigator or sub-investigator determines that it is necessary, the following treatments may be administered.

- ✓ VEGF inhibitor
- ✓ Intravitreal gas injection
- ✓ Photodynamic therapy

6.2. Preparation, Handling, Storage, and Management

1) The investigational product may be administered only to subjects, and only the principal investigator, sub-investigator, or other personnel pre-designated in advance may prepare and administer the investigational product.

2) Procedures and records concerning receipt, inventory entry, dispensing, and disposal of the investigational product shall be performed in accordance with the separately defined "Standard Operating Procedure for Investigational Product Management."

6.3. Allocation of Trial Intervention

The principal investigator or sub-investigator shall, after obtaining written informed consent from the patient, confirm that all inclusion criteria are met and that none of the exclusion criteria apply. Thereafter, the principal investigator, sub-investigator, or clinical research coordinator will perform registration using an electronic data capture (EDC) system. All registered subjects will be assigned to the active-drug group.

[EDC System]

Fountayn

Registration hours: 24-hour registration possible (excluding maintenance/inspection periods)

Contact: Kyushu University Hospital ARO Next-Generation Medical Center, Clinical Research Management Division, Data Science Office

Weekdays 9:00 – 17:00 (excluding Saturdays, Sundays, public holidays, and the year-end/New-Year period (12/29 – 1/3))

Precautions for registration:

- ✓ Registration after administration of monteplase (recombinant) is not permitted under any circumstances.
- ✓ In the EDC system, registration will not be accepted until all required input fields are completed.
- ✓ Registration is considered complete when eligibility is confirmed and a registration number is issued.
- ✓ Except when a subject withdraws consent, including refusal to allow data use, a subject once registered will not have that registration cancelled. In the event of duplicate registration, the information from the first registration (including registration number) will be used.
- ✓ If erroneous or duplicate registration is discovered, the data-management staff must be notified promptly.

6.4. Blinding

No blinding will be performed.

6.5. Compliance with Trial Intervention

Under medical management, the subject shall receive the trial intervention from the principal investigator, sub-investigator, or a pre-designated responsible individual. The date of intervention, the eye receiving monteplase (recombinant), the administered dose, the injection site, the details of positional restriction, and, if the dose is reduced or increased from the stipulated amount (0.1 mL), the reason shall be recorded; for gas tamponade or silicone oil exchange, the material used shall be recorded.

6.6. Dose Adjustment of Trial Intervention

If, based on the extent of hemorrhage or retinal condition, sub-retinal administration of 0.1 mL is judged to be difficult (e.g., investigational product spreading beyond the retinal vascular arcade) by the principal investigator or sub-investigator, the dose may be reduced as appropriate. Furthermore, after Visit 1, no additional sub-retinal administrations shall be performed except in cases of re-bleeding.

6.7. Continuation of Trial Intervention After Trial Completion

No specific treatment after completion of this study will be stipulated. However, for subjects who provide consent to participate in an observational study for long-term visual prognosis, ophthalmologic examination results will continue to be collected after completion of the study.

6.8. Treatment of Overdose

Administration of recombinant monteplase exceeding 8,000 IU shall be considered an overdose. In the event of an overdose, the principal investigator or sub-investigator shall take the following actions:

1) Observe the subject's condition and provide medically necessary interventions.
2) Carefully monitor the subject for any adverse events and any abnormal clinical laboratory values.
3) Record the amount of the overdose and report it, together with the subject's condition, to the trial-coordinating physician.
4) Document the reason for the overdose and the administered dose in the case report form.

6.9. Prior Treatment and Concomitant Therapy

6.9.1. Prior Treatment

If, prior to participation in this trial, the subject has received any of the following treatments for the current sub-macular hemorrhage, the date of treatment, details of the treatment (product name and/or type of gas), and dose shall be recorded in the case report form.

i. Intravitreal administration of a tPA product.
ii. Intravitreal gas injection.

6.9.2. Concomitant Medication / Concomitant Therapy

6.9.2.1. Prohibited Medications / Prohibited Therapies

The following are prohibited during the study period:
1) Defibrinated sodium (Defaiterio).
2) Intravenous, sub-retinal, or intravitreal administration of tPA products other than monteplase (recombinant).
3) Intravenous or intravitreal administration of monteplase (recombinant).
4) Ophthalmic surgeries not listed in 6.9.2.2.

6.9.2.2. Permitted Medications / Permitted Therapies

The following surgical treatments or drug uses are permitted:
1) Phacoemulsification cataract extraction and intra-ocular lens implantation.
2) Epiretinal membrane and internal limiting membrane peeling.

3) Retinal photocoagulation or cryotherapy for retinal tears.
4) Antiplatelet agents (including agents with platelet-aggregation-inhibiting activity) and anticoagulants.

7. Discontinuation of Trial Intervention and Subject Discontinuation/Withdrawal
This section describes discontinuation for individual subjects; discontinuation of the trial at a trial site or termination of the entire trial is described in Appendix 1.

7.1. Discontinuation of Trial Intervention
If, prior to surgery, the principal investigator or sub-investigator determines that the subject's systemic condition has deteriorated such that sub-retinal administration of monteplase (recombinant) cannot be performed, the trial intervention shall be discontinued.

7.2. Subject Discontinuation/Withdrawal
The trial for a subject shall be discontinued if any of the following applies:
1) The subject withdraws consent (the detailed reason for withdrawal shall be recorded in the case report form).
2) The subject is found to be ineligible after enrollment.
3) Based on a comprehensive safety assessment, including the occurrence of adverse events, the principal investigator or sub-investigator judges that continuation of the trial is infeasible (the detailed reason for infeasibility shall be recorded in the case report form).
4) The subject dies.
5) After trial initiation it becomes evident that, due to the subject's circumstances, required subsequent examinations or observations cannot be performed.
6) The subject meets the definition of loss to follow-up as defined in Section 7.3.
7) Any other situation in which the principal investigator or sub-investigator judges continuation of the trial to be infeasible (the detailed reason for trial discontinuation shall be recorded in the case report form).

At the time of discontinuation, appropriate medical care shall be provided based on the physician's judgment. To the extent feasible, the observations and examinations specified in the protocol for trial discontinuation shall be performed to evaluate safety. If the trial is discontinued because of an adverse event, the event shall be classified according to Section 10.2.3 (outcome categories 1, 4, 5) and follow-up of the adverse event shall be conducted up to 28 days after discontinuation. If follow-up cannot be performed, the reason shall be documented in the case report form. No discontinuation assessments shall be performed

for discontinuations that occur prior to any trial intervention.

A subject may withdraw from the trial at any time, for any reason or without providing a reason, upon the subject's own request, and no disadvantage shall result. Even if a subject withdraws consent, data collected prior to withdrawal shall, in principle, be included in the analysis set.

7.3. Loss to Follow-up

A subject shall be considered lost to follow-up if the subject fails to attend scheduled visits and the trial site is unable to contact the subject.

If a subject does not attend a scheduled visit, the following actions shall be taken:

1) The trial site will contact the subject and reschedule the visit as soon as possible for any assessment date on which the subject did not attend the visit. Explain to the subject the importance of adhering to the instructed visit date, and confirm whether the subject wishes to continue participation in the trial and whether continuation is appropriate.

2) Before determining that a subject is lost to follow-up, the principal investigator, sub-investigator, or trial collaborator shall make every effort to contact the subject (by telephone or, if necessary, by sending certified mail to the last known address of the subject). The details of these actions shall be recorded in the subject's medical record.

3) If the inability to contact the subject persists, the subject shall be considered to have withdrawn from the trial.

8. Trial Assessments and Procedures

- ✓ The timing of trial evaluations is described in Section 1.3 Schedule. The procedures described in this section and the Schedule in Section 1.3 must be adhered to.
- ✓ Data obtained as part of routine clinical care and collected prior to obtaining informed consent for this trial may be used as screening or baseline data provided that they meet the criteria specified in the protocol and were performed within the time windows defined in the Schedule of Section 1.3.
- ✓ If a serious problem affecting trial continuation arises (e.g., a pandemic), alternative methods for subject visits, assessments, and monitoring may be implemented in accordance with regulatory authority and ethics committee requirements.
- ✓ For safety reasons or technical issues related to assessments, re-evaluation or off-schedule evaluations may be performed.

8.1. Screening (Baseline) Assessment Procedures

The procedures for ophthalmic examinations are described in 8.2 Efficacy Evaluation

Procedures.

8.1.1. Subject Background

The following information shall be collected during the screening period.

- Date of informed consent
- Sex
- Age at the time of informed consent
- Date of diagnosis of AMD or RAM and treatment history
- Medical history (ocular diseases, and only those deemed clinically significant for this trial by the principal investigator or sub-investigator shall be recorded in the case report form)
- Comorbidities
- Presence or absence of drug allergy
- Prior treatment (if present, collect the information described in Section 6.9.1)

8.1.2. Height and Weight

Measure height and weight.

8.1.3. Chest X-ray

Obtain a frontal chest X-ray in the standing position. If standing is difficult, a seated position is acceptable. Record the presence or absence of abnormalities, and if present, document the findings in the case report form.

8.1.4. Electrocardiogram (ECG)

Perform a resting 12-lead electrocardiogram. Record the presence or absence of abnormalities, and if present, document the findings in the case report form.

8.2. Efficacy Assessment Procedures

8.2.1. Optical Coherence Tomography (OCT) (both eyes)

Using OCT under mydriasis, capture fundus images and measure CFT (μm). Enter the measurement results and the instrument used in the case report form. When measurements are performed at the same medical institution, the same device shall be used throughout the clinical trial period, in principle.

8.2.2. Fundus Photography (both eyes)

Under pharmacologic dilation, capture fundus photographs and assess for the presence of a

central foveal hemorrhage ≥ one optic-disc diameter, as well as for marked organization or fibrosis of the hemorrhage. Document any other abnormalities in detail in the case report form.

8.2.3. Visual Acuity Testing (both eyes)

Measure BCVA using the international Landolt C chart, and record the decimal visual acuity and the logMAR conversion value in the case report form. For partial visual acuity, enter the converted value in the case report form according to the conversion table in Table 8-1. If visual acuity cannot be measured with the Landolt C chart, document the measurement method and the result in the case report form.

Table 8-1　Conversion of Partial Visual Acuity Test Results

Test Result	Conversion Value
2.0p	1.5
1.5p	1.2
1.2p	1.0
1.0p	0.9
0.9p	0.8
0.8p	0.7
0.7p	0.6
0.6p	0.5
0.5p	0.4
0.4p	0.3
0.3p	0.2
0.2p	0.1
0.1p	0.09
0.09p	0.08
0.08p	0.07
0.07p	0.06
0.06p	0.05
0.05p	0.04
0.04p	0.03
0.03p	0.02
0.02p	0.01
0.01p (CF)	0.004
HM	0.002

SL+	0.001

8.2.4. Intraocular Pressure Measurement (Visit 2/3/4: treated eye only; other visits: both eyes)

Intraocular pressure shall be measured using a Goldmann applanation tonometer or an automated tonometer (non-contact tonometer, iCare handheld tonometer, Tonopen). The measurement result and the device used shall be entered into the case report form.

8.2.5. Anterior Segment Examination (Visits 2/3/4: treated eye only; other visits: both eyes)

Under pharmacologic dilation, the items listed in Table 8-2 shall be examined with a slit-lamp microscope and the presence or absence of each finding shall be recorded. If examination is not feasible, the reason shall be documented in the case report form.

Table 8-2 Slit-lamp Examination Grading Criteria

Observation Items		Grade	Grading Criteria
Eyelid	Erythema	None<br>Mild<br>Moderate<br>Severe	No erythema or only minimal erythema<br>Localized conjunctival vessel dilation<br>Partial conjunctival vessel dilation<br>Conjunctival vessels so dilated that individual vessels are indistinguishable
	Edema	None<br>Mild<br>Moderate<br>Severe	No edema or only minimal edema<br>Focal swelling<br>Diffuse mild swelling<br>Cystoid edema
Conjunctiva	Hyperemia	None<br>Mild<br>Moderate<br>Severe	No hyperemia or only minimal<br>Partial conjunctival vessel dilation (limited area)<br>Partial conjunctival vessel dilation<br>Diffuse conjunctival vessel dilation
	Edema	None<br>Mild<br>Moderate<br>Severe	No edema or only minimal<br>Partial swelling<br>Diffuse mild swelling<br>Cystoid edema
	Hemorrhage	None<br>Mild<br>Moderate<br>Severe	No hemorrhage or only minimal<br>Partial hemorrhage<br>Hemorrhage present but not circumferential<br>Circumferential hemorrhage

Cornea	Edema	None<br>Mild<br>Moderate<br>Severe	No edema or only minimal<br>Partial swelling<br>Diffuse mild swelling<br>Cystoid edema
Anterior Chamber	Cell Count	None<br>Mild<br>Moderate<br>Severe	≤ 5 cells<br>6 – 25 cells<br>26 – 50 cells<br>> 50 cells
	Flare	None<br>Mild<br>Moderate<br>Severe	No protein or only trace<br>Opacity present but iris pattern clearly observable<br>Opacity makes iris pattern observation somewhat difficult<br>Marked opacity or fibrin deposition
Iris	Abnormal Findings	None<br>Mild<br>Moderate<br>Severe	No abnormality or only very mild abnormality<br>Mild abnormality<br>Moderate abnormality<br>Severe abnormality
Vitreous	Opacity	None<br>Mild<br>Moderate<br>Severe	No abnormality<br>Mild opacity (optic disc and vessels observable)<br>Moderately severe opacity (optic disc faintly observable)<br>Fundus not visualizable at all
	Hemorrhage	None<br>Mild<br>Moderate<br>Severe	No hemorrhage<br>Mild hemorrhage (optic disc and vessels observable)<br>Moderately severe hemorrhage (optic disc faintly observable)<br>Fundus not visualizable at all
Lens	Presence	None<br>Intra-ocular lens<br>Natural lens present	aphakic eye<br>pseudophakic eye<br>phakic eye
	Opacity	None	No opacity

		Mild	Mild opacity (optic disc and vessels observable)
		Moderate	Moderately severe opacity (optic disc faintly observable)
		Severe	Fundus not visualizable at all

8.2.6. Fundus Examination (Visits 2/4: treated eye only; other visits: both eyes)

Under mydriasis, perform indirect ophthalmoscopy or slit-lamp biomicroscopy to assess the presence or absence of macular hemorrhage. Any other abnormalities shall be described in detail in the case report form. If the fundus cannot be visualized or is indeterminate because of vitreous hemorrhage, vitreous opacity, etc., or if the examination is difficult to perform, the reason shall be recorded.

8.2.7. Concomitant Medications / Concomitant Therapies

Concomitant medications shall be recorded in the case report form with the drug name, start date, end date, route of administration, reason for concomitant use, and, for ophthalmic medications, the eye(s) to which the medication is administered.

Documentation of anesthetic agents, disinfectants, antibiotics, etc., used during surgery, and mydriatic agents used during examinations are not required.

Concomitant therapies shall be recorded with the therapy name, start date, end date, reason for concomitant use, and, for therapies directed to the eye, the target eye shall be entered in the case report form.

However, if a tPA preparation is administered sub-retinally at the time of re-bleeding, in addition to the above, the administered dose, type of replacement procedure, replacement material, and details of positional restrictions shall also be recorded.

8.3. Safety Assessment Procedures

8.3.1. Vital Signs

Blood pressure (systolic/diastolic) and pulse rate shall be measured using an automated sphygmomanometer while the subject is seated or supine at rest. Axillary body temperature shall also be measured.

On Day 1, measurements shall be taken before and after administration of the investigation product.

8.3.2. Laboratory Blood Tests

The following parameters shall be measured.

[Hematology] Red blood cell count, hemoglobin, hematocrit, white blood cell count, differential white blood cell count (neutrophils, lymphocytes, monocytes, eosinophils, basophils), platelet count

[Clinical Chemistry] Albumin, total bilirubin, direct bilirubin, AST, ALT, alkaline phosphatase, LDH, γ-GTP, BUN, creatinine, CRP, sodium, potassium, chloride, calcium, glucose (as needed)

[Coagulation] PT-INR, APTT, fibrinogen, D-dimer

8.3.3. Urinalysis

Urine protein and urine occult blood shall be examined qualitatively.

8.3.4. Clinical Laboratory Evaluations

The principal investigator or sub-investigator shall, based on clinical laboratory results, report any clinically significant changes occurring during the trial as adverse events. Examples of changes considered clinically significant are listed below.

- ✓ When accompanied by symptoms (however, laboratory abnormalities related to the underlying disease shall not be deemed clinically significant unless the principal investigator or sub-investigator judges that they are more severe than would be expected based on the subject's condition).
- ✓ When additional testing is required, or when medical or surgical intervention is required.
- ✓ If the trial is to be discontinued due to abnormal laboratory values
- ✓ If the principal investigator or sub-investigator determines that an adverse event has occurred

All clinical laboratory values that are considered clinically significant abnormalities during trial participation shall be repeated until they return to normal values or baseline, or until the principal investigator or sub-investigator no longer considers them clinically significant. If clinically important values do not return to normal/baseline within the period deemed appropriate by the principal investigator or sub-investigator and follow-up is terminated, the reason shall be recorded in the medical record.

8.3.5. Pregnancy Testing

(Only for women of child-bearing potential) Pregnancy test (urine hCG)

8.4. Adverse Events, Serious Adverse Events, and Other Safety Reporting

The definitions of adverse event (AE) and serious adverse event (SAE) are provided in

Appendices 2-10.2.1 and 10.2.2. The principal investigator or sub-investigator is responsible for the identification, documentation, and reporting of any event that meets the definitions of AE or SAE. They are also responsible for the follow-up of all adverse events (see Section 7.2). This includes events reported on the subject. The procedures for recording, assessing, determining causality of AEs or SAEs, and for preparing and transmitting SAE reports are described in Appendix 2-10.2.3.

8.4.1. Collection Period and Frequency for Adverse Events and Serious Adverse Events

The collection period for all AEs and SAEs shall be from the first administration of the investigational product until the end of the observation period, as specified in Section 1.3 Clinical Trial Schedule. Events that occur after informed consent are obtained but before the first dose of the investigational product shall be recorded as medical history/comorbidities, not as AEs. All SAEs shall be recorded promptly in accordance with Appendix 2-10.2.4 and reported to the trial coordinating physician, in principle, within 24 hours regardless of the circumstances.

The principal investigator and sub-investigator are not obligated to actively collect information on AEs or SAEs that occur after the above-specified period. However, if an SAE occurring after the period is known for an individual subject and is considered to have a reasonable causal relationship with the investigational product or trial participation, the principal investigator or sub-investigator shall promptly notify the trial coordinating physician.

For the collection period of pregnancy information and, when applicable, the follow-up period for pregnancy, see Section 8.4.5.

8.4.2. Identification of Adverse Events and Serious Adverse Events

When identifying AEs and SAEs, care shall be taken to avoid bias, for example by asking open-ended questions to the subject regarding the occurrence of events.

8.4.3. Follow-up of Adverse Events and Serious Adverse Events

After the initial AE/SAE report, the principal investigator or sub-investigator shall actively follow each subject at subsequent visits or contacts. All AEs shall be followed until the event resolves, the condition stabilizes, it becomes evident that the event was caused by another factor, or the subject becomes lost to follow-up (as defined in 7.3). Details of the follow-up procedures are provided in Appendix 2-10.2.3.

8.4.4. Regulatory Reporting Requirements for Serious Adverse Events

The principal investigator or sub-investigator shall, in order to fulfill their legal obligations and

ethical responsibilities for subject safety and the safety of the trial intervention, promptly initiate the reporting process for an SAE in accordance with Appendix 2-10.2.4 and the "Procedures for Handling Safety Information." In addition, the trial coordinator shall, in accordance with Appendix 2-10.2.4 and the "Procedures for Handling Safety Information," make reports to the Pharmaceuticals and Medical Devices Agency as required to satisfy regulatory obligations.

8.4.5. Pregnancy Information

Details of all pregnancies occurring in women of child-bearing potential shall be tracked from the time of investigational product administration until the end of the observation period in accordance with the following procedures.

- ✓ If a pregnancy is reported, the principal investigator shall record the pregnancy information on the appropriate form and submit it promptly to the trial coordinator upon becoming aware of the subject's pregnancy.
- ✓ Pregnancy itself is not considered an AE or SAE; however, pregnancy complications or elective termination shall be reported as an AE or SAE.
- ✓ If the outcome of the pregnancy is abnormal (e.g., spontaneous abortion, fetal death, stillbirth, congenital anomaly, ectopic pregnancy), it shall be reported as an SAE.
- ✓ The subject shall be followed to ascertain the pregnancy outcome. The principal investigator or sub-investigator shall collect follow-up information concerning the subject and the neonate and report it to the trial coordinator.
- ✓ The principal investigator or sub-investigator shall, if an SAE related to pregnancy occurs after trial completion and a reasonable possibility of a causal relationship with the investigational product is judged, report it to the trial coordinator in accordance with this section. The principal investigator and sub-investigator are not obligated to actively collect such information after a subject has discontinued trial participation, although they may become aware of an SAE through spontaneous reporting.
- ✓ Evaluation of the disease condition for participants who become pregnant during the trial may be continued if the principal investigator or sub-investigator deems it appropriate.

8.4.6. Cardiovascular Events and Deaths

No cardiovascular events of particular concern are anticipated in this trial.

8.4.7. Disease-Related Events

Events that may occur in association with the underlying disease or the surgical procedure include pre-retinal hemorrhage, intraretinal hemorrhage, sub-retinal hemorrhage, vitreous hemorrhage, serous retinal detachment, macular edema, re-bleeding, hard exudates, etc. These

events shall be evaluated, in light of the subject's underlying disease condition, for the anticipated intensity, frequency, duration, and any reasonable possibility of association with the trial intervention, to determine their relevance as an AE or SAE and to assess causality with the investigational product.

8.5. Pharmacokinetics (PK)

Pharmacokinetic assessments will not be performed in this trial.

8.6. Pharmacodynamics

Pharmacodynamic assessments will not be performed in this trial.

8.7. Genetics

Genetic assessments will not be performed in this trial.

8.8. Biomarkers

Biomarker assessments will not be performed in this trial.

8.9. Immunogenicity Assessment

Immunogenicity assessments will not be performed in this trial.

8.10. Utilization of Medical Resources and Health Economics

Health-economic analyses, utilization of medical resources, and health-economic parameters will not be evaluated in this trial.

9. Statistical Considerations

The statistical analysis plan will be finalized prior to database lock and will contain a more technical and detailed description of the statistical analyses described in this section. This section provides a summary of the planned statistical analyses for the most important endpoints, including primary and secondary endpoints.

9.1. General Considerations

- Baseline Definition

Baseline assessments or events are defined as assessments or events that occurred prior to the date and time of administration of the investigational product. When multiple valid assessments or events are available, the assessment closest to the investigational product administration date (and time, if collected) shall be used as the baseline for analysis.

- Summary Statistics

For continuous variables, summary statistics shall include the number of observations, mean, standard deviation, minimum, median, maximum, first quartile (25th percentile), and third quartile (75th percentile). For categorical variables, summary statistics shall include the number of observations and the frequency and percentage (%) of each category.

Means, standard deviations, medians, first quartiles, and third quartiles shall be rounded to two decimal places and displayed to one decimal place. Minimum and maximum values shall be rounded to one decimal place and displayed to the specified number of digits. Percentages (%) and the upper and lower limits of their confidence intervals shall be rounded to the second decimal place and displayed to one decimal place.

- Significance Level and Confidence Coefficient

Unless otherwise specified, the significance level for hypothesis testing shall be two-sided 5 %. For interval estimation, unless otherwise stated, two-sided intervals shall be used with a confidence coefficient of 95 %. P-values shall be rounded to the fourth decimal place and displayed to three decimal places; however, if the unrounded p-value is less than 0.001, it shall be uniformly reported as ″<0.001″.

9.1.1. Statistical Hypotheses

The primary objective is to evaluate the efficacy of sub-retinal administration of Monteplase (recombinant) for foveal hemorrhage in patients with SMH secondary to AMD or RAM.

For the primary endpoint—the change from baseline in CFT at Week 1—the 95 % confidence interval of the mean change shall be calculated, and a t-test shall be performed to assess statistical significance.

Accordingly, the null hypothesis to be tested for the primary endpoint is:

Null hypothesis: The mean change from baseline in CFT at Week 1 is 0.

Alternative hypothesis: The mean change from baseline in CFT at Week 1 is not 0.

9.1.2. Adjustment for Multiplicity

The principal analysis for this trial is the t-test of the change from baseline in CFT at Week 1 performed on the Full Analysis Set (FAS). In principle, no adjustment for multiplicity will be applied to either the primary or secondary endpoints.

9.1.3. Impact of Strategy for Intercurrent Events

Table 3-1 lists the interim events anticipated at the start of the trial. Considering the objectives of the trial, intercurrent events that are unlikely to represent clinical treatment failure, or for which the treatment strategy would be prioritized regardless of their occurrence, are handled

using a treatment policy strategy. Under the treatment policy strategy, all observed data regardless of the occurrence of intercurrent events will be included in the analysis.
Conversely, intercurrent events that are not aligned with the objectives of the trial are handled using a hypothetical strategy. Under the hypothetical strategy, the treatment effect is defined as if the intercurrent event had not occurred. To estimate this effect, data collected after the occurrence of the intercurrent event will be excluded from the analysis.
Furthermore, intercurrent events that are considered to represent clinical treatment failure are handled using a composite strategy. Under the composite strategy, the occurrence of the intercurrent event is incorporated into the endpoint definition. In this trial, a value representing no improvement will be assigned to such cases, and the analysis will be performed accordingly.

9.1.4. Handling of Missing Data
The handling of missing data shall be described in detail in the Statistical Analysis Plan.
Except as specified in the Statistical Analysis Plan, missing values shall not be imputed as a general principle.

9.2. Analysis Populations
For the purpose of analysis, the following analysis populations are defined.

Analysis Population	Definition
Informed Consent Population	All subjects who provided informed consent.
Enrolled Population	All subjects who were formally enrolled.
Intervention Population	Subjects among the enrolled cohort who received the investigational drug. This group constitutes the primary safety analysis set.
Intervention Discontinuation Population	Subjects among the enrolled cohort whose investigational drug administration was discontinued.
Completion Population	Subjects from the trial-intervention population who completed the treatment period and observation period and attended a visit at week 12 (week 12 ± 7 days).
Discontinuation Population	Subjects from the trial-intervention population whose follow-up during the treatment period or observation period was discontinued and who did not attend a visit at week 12 (week 12 ± 7 days).
FAS (Full Analysis Set)	From the Intervention Cases, subjects meeting any of the following criteria are excluded.<br>● No efficacy data exists after investigational drug

	administration.<br>● Serious GCP violation.

9.3. Analyses Supporting the Primary Objective

9.3.1. Primary Endpoint

The primary endpoint shall be the change from baseline in CFT at week 1.

9.3.1.1. Definition of the Endpoint

- Change: The change from baseline shall be calculated using the following formula.
  Change = measurement at week 1 − baseline value

9.3.2. Primary Analysis Method

For CFT, the 95 % confidence interval of the mean change from baseline at week 1 will be calculated, and a significance test will be performed using a t-test.

Intercurrent events shall be handled as follows.

For vitreous re-surgery for ocular-complication treatment, sub-Tenon injection of triamcinolone acetonide, rescue therapy with photodynamic therapy or VEGF inhibitors, and the administration of prohibited concomitant therapy for other diseases such as defibrotide sodium or tPA preparations (including monteplase) (adopting a treatment policy strategy approach), all observed values of the primary endpoint shall be used in the analysis. For withdrawal of consent or loss to follow-up not due to serious adverse events, intravitreal gas injection as rescue therapy, and the administration of prohibited tPA preparations (including monteplase) as additional treatment for sub-macular hemorrhage (adopting a hypothetical strategy approach), data collected after the occurrence of the intercurrent event shall be excluded from the analysis in order to evaluate the treatment effect without these influences. For vitreous re-surgery due to re-bleeding, withdrawal of consent due to serious adverse events, and hematoma-removal surgery using prohibited concomitant therapy (adopting a composite strategy), the change value of 0 shall be imputed as the worst value for the primary endpoint in cases where the intercurrent event occurred, and these cases shall be analyzed accordingly.

9.3.2.1. Sensitivity Analysis

For CFT, the significance of the change from baseline at week 1 shall be tested using the Wilcoxon signed-rank test.

The Wilcoxon signed-rank test is a non-parametric test that does not assume the change follows a normal distribution and will be performed as a sensitivity analysis.

9.3.2.2. Supplemental Analysis

In addition to the primary and sensitivity analyses, for cases in which intercurrent events occur multiple times or serious adverse events occur multiple times, the number of occurrences will be identified and the relationship with the primary endpoint will be analyzed.

Furthermore, considering the pure pharmacologic effect of the investigational product after excluding the influence of VEGF-inhibitor use or tPA-preparation use for other diseases, and recognizing that this is important for the treating physician's clinical decision-making for each patient, an evaluation will be performed assuming a virtual strategy in which the intercurrent events of VEGF-inhibitor use and the use of monteplase or other tPA preparations for other diseases are assumed not to have occurred. Specifically, data collected after the occurrence of the intercurrent event will be excluded, and the analysis will be conducted in the same manner as the primary analysis.

9.4. Analyses Supporting Secondary Objectives

9.4.1. Analyses Supporting Secondary Objectives

1) Change from baseline in CFT at week 4

For CFT, the 95 % confidence interval of the mean change from baseline at week 4 will be calculated, and a significance test will be performed using a t-test.

2) Presence of a foveal hemorrhage ≥1 disc diameter at week 4

The proportion of subjects with a foveal hemorrhage ≥1 disc diameter at week 4 will be evaluated for significance using a chi-square test.

3) BCVA (logMAR equivalent) at week 4

For BCVA (logMAR equivalent), the 95 % confidence interval of the mean at week 4 will be calculated.

4) Change from baseline in BCVA (logMAR equivalent) at week 4

For BCVA (logMAR equivalent), the 95 % confidence interval of the mean change from baseline at week 4 will be calculated, and significance will be tested using a t-test.

5) Change from baseline in CFT at week 12

For CFT at week 12, the 95 % confidence interval of the mean change from baseline will be calculated, and significance will be tested using a t-test.

6) BCVA (logMAR equivalent) at week 12

For BCVA (logMAR equivalent), the 95 % confidence interval of the mean at week 12 will be calculated.

7) Change from baseline in BCVA (logMAR equivalent) at week 12

For BCVA (logMAR equivalent), the 95 % confidence interval of the mean change from baseline at week 12 will be calculated, and significance will be tested using a t-test.

9.5. Exploratory Objective Analyses

Not applicable.

9.6. Safety Analyses

For the following items, the number of subjects evaluated, the number of events, and the incidence proportion will be calculated.

- Adverse events, adverse drug reactions
- Serious adverse events, serious adverse drug reactions
- Adverse events resulting in death, adverse drug reactions resulting in death
- Adverse events leading to discontinuation of the trial, adverse drug reactions leading to discontinuation of the trial

9.7. Other Analyses

9.7.1. Other Variables and/or Parameters

Not applicable.

9.7.2. Subgroup Analysis

Subgroup analyses will be performed for the following factors and categories. In the subgroup analyses, the same analyses as the primary analysis will be conducted.

Factor Name	Category
Cause of sub-macular hemorrhage	AMD, RAM
Dose of tPA formulation	≤ 8,000 IU, > 8,000 IU

9.8. Interim Analysis

No interim analysis is planned for this clinical trial.

9.9. Determination of Sample Size

- Target number of subjects: 20 (however, a minimum of three subjects must be enrolled for each disease, AMD and RAM)

Rationale for the target: In a retrospective review of our institution over the past approximately two years, eight patients (11 eyes) who received sub-retinal tPA for SMH with a pre-treatment CFT ≥ 300 μm were identified. The change in CFT at one week post-procedure had a mean of −109.36 μm and a standard deviation of 176.98 μm (unpublished data). The corresponding

effect size d was 0.62 (effect size d = mean difference / SD). Because SMH patients meet the criteria for a rare disease and because this trial will enroll patients with at least a moderate amount of hemorrhage and at least a moderate visual-acuity loss, a modest clinical improvement is anticipated. Assuming an effect size of 0.7 (mean change −120 µm, SD 170 µm), a two-sided α of 5 % and 80 % power, the required sample size was calculated to be 19 subjects. Allowing for a 5 % dropout rate, the total target sample size was set at 20 subjects.

10. Appendix

Appendix 1:

10.1. General Considerations Regarding Regulation, Ethics, and Trial Management

10.1.1. Regulatory and Ethical Considerations

This clinical trial will be conducted in compliance with the following laws, regulations, and standards:

- ✓ The ethical principles of the Declaration of Helsinki (1964) and its revisions
- ✓ The standards stipulated in Article 14, Paragraph 3 and Article 80-2 of the Pharmaceuticals and Medical Devices Act
- ✓ The "Ordinance on Standards for Conducting Clinical Trials of Pharmaceuticals (Good Clinical Practice, GCP)" (Ministry of Health, Labour and Welfare Ordinance No. 28 of 27 March 1997) and its amended ordinances and related guidance
- ✓ Standard Operating Procedures
- ✓ This clinical trial protocol

10.1.2. Compliance with the Trial Protocol, Deviations, and Amendments

10.1.2.1. Compliance with the Clinical Trial Protocol

The principal investigator shall submit the clinical trial protocol and GCP-required documents such as the explanatory material and informed-consent form to the head of the trial site and obtain written approval for conducting the trial. The principal investigator, sub-investigators, and trial collaborators shall conduct the trial in accordance with the clinical trial protocol.

10.1.2.2. Deviations from the Clinical Trial Protocol

Except in the following cases, the principal investigator or sub-investigator shall not deviate from or amend the clinical trial protocol without obtaining prior written approval based on review by the Institutional Review Board (IRB):

1) When it is medically unavoidable, such as to avoid an imminent danger to a subject;

2) When the change concerns only administrative matters of the trial.

In case of 1), the principal investigator shall, as soon as possible, submit to the head of the trial site and the IRB the details and reasons for the deviation or amendment, and, if appropriate, a draft of the revised protocol, and obtain approval.

The principal investigator shall document and retain in writing all actions that deviate from the

clinical trial protocol.

10.1.3. Amendments to the Clinical Trial Protocol

If the principal investigator determines that amendment of the clinical trial protocol is necessary, or if the IRB directs an amendment, the trial coordinating physician shall amend the protocol. The principal investigator shall report the amendment content and its rationale to the head of the trial site and obtain IRB approval.

The amendment procedure shall follow the "Procedures for Preparation of the Clinical Trial Protocol."

10.1.4. Institutional Review Board

10.1.4.1. Review of Clinical Trial Conduct

The appropriateness of conducting this trial shall be reviewed by the Institutional Review Board from ethical, scientific, and medical/pharmacological perspectives. The trial will be conducted only after obtaining IRB approval. If the IRB directs any modifications to the informed-consent document or other materials, the principal investigator at the respective medical institution shall address them.

10.1.4.2. Continuing Review

The principal investigator shall submit, in writing to the head of the trial site, a summary of the current status of the trial at least annually, or more frequently as requested by the IRB, in order to undergo the IRB's continuing review. The head of the trial site shall obtain the IRB's opinion regarding the appropriateness of continuing the trial at that site.

10.1.5. Procedures for Explanation and Obtaining Informed Consent

10.1.5.1. Preparation of the Explanation Document and Informed Consent Form

The principal investigator shall prepare the explanation document and the informed consent form and obtain prior approval from the IRB. The items to be included in the explanation document shall be prepared in accordance with Article 51 of the Ordinance on Standards for Conducting Clinical Trials of Pharmaceuticals and its accompanying guidance.

10.1.5.2. Obtaining Consent

The principal investigator or sub-investigator shall, prior to a patient's participation in the trial, provide a thorough explanation using the explanation document, confirm that the patient has

fully understood the content, and obtain the patient's voluntary written consent to participate. The patient who has received the explanation shall, after fully understanding the content of the explanation document, sign or affix a seal to the informed consent form indicating agreement to participate in the trial and date it. The principal investigator or sub-investigator who provided the explanation shall, after confirming the patient's comprehension of the explanation document, also sign or affix a seal to the same informed consent form and date it. If a trial collaborator provides supplemental explanation, that collaborator shall likewise sign or affix a seal and date the form.

The principal investigator or sub-investigator shall deliver the signed informed consent form and the explanation document to the subject and shall retain the original informed consent form in the medical institution's records.

10.1.5.3. Notification to Other Institutions at the Time of Consent

The principal investigator or sub-investigator shall ascertain whether the subject is receiving care at another hospital or department; if so, after obtaining the subject's consent, the investigator shall notify the subject's attending physician of the subject's participation in this trial.

10.1.5.4. Points to Consider When Obtaining Consent

When obtaining consent, the following points shall be observed.

- ✓ The principal investigator or sub-investigator shall explain the trial using plain language that enables the patient to understand the study details.
- ✓ The principal investigator or sub-investigator must not coerce the patient to participate in or continue the study, nor exert undue influence on the patient's decision.
- ✓ The principal investigator or sub-investigator shall, after providing the explanation, give the patient an opportunity to ask questions, continue the explanation until the patient is satisfied, and allow sufficient time for the patient to decide on participation.
- ✓ The principal investigator or sub-investigator shall specify the visit date to the patient, explain that investigations/observations will be performed, and obtain the patient's consent.
- ✓ In the explanation, no language should be used that would cause the subject to waive or doubt his/her rights, or that would exempt or cast doubt on the legal responsibility of the principal investigator or the trial site.
- ✓ In this clinical trial, obtaining consent through a proxy is not permitted.
- ✓ If a subject has difficulty reading the informed-consent document because of reduced visual acuity, visual field, or other visual-function impairment, a neutral witness shall be required during the explanation to the subject. A neutral witness must be independent of

the conduct of this trial and must not be unduly influenced by persons involved in the trial.

- ✓ Subjects undergoing rescreening must sign a new informed-consent document.

10.1.5.5. Revision of the Informed-Consent Document

If new information is obtained that may affect a subject's decision to continue participation in the trial, the principal investigator or sub-investigator shall promptly convey this information to the subject, confirm the subject's intention regarding continued participation, and record it. The principal investigator shall also promptly revise the informed-consent document based on the new information, submit the revised document to the head of the trial site, and obtain approval from the Institutional Review Board. The principal investigator or sub-investigator shall use the revised informed-consent document to provide a thorough explanation to the subject, reconfirm the subject's intention to continue participation, and obtain written consent.

10.1.6. Compensation for Health Injuries

The sponsor and the trial site shall secure measures to fulfill compensation and indemnification obligations for health injuries to subjects, such as obtaining clinical-trial insurance and other necessary actions.

If a subject experiences a health injury as a result of participation in this trial, the trial site shall provide necessary and appropriate medical care and other measures for the injury. For health injuries attributable to this trial, compensation, medical expenses, and medical allowances shall be paid in accordance with the terms of the clinical-trial insurance. However, compensation shall not be provided, in principle, for failure to achieve the expected pharmacologic effect or other benefits of the investigational product.

Health injuries arising from the following reasons shall not be compensated or shall result in a reduction of compensation:

- ✓ When caused by the subject's intentional act or gross negligence.
- ✓ When caused by the principal investigator's or sub-investigator's serious deviation from the clinical-trial protocol, or by the trial site's intentional act or gross negligence.
- ✓ When caused by illegal acts or non-performance of a third party.
- ✓ When caused by chance events (e.g., being struck by a reckless vehicle while traveling to the clinic, food poisoning, etc.).

10.1.7. Strategies for Subject Recruitment

To facilitate broad participant recruitment in this study, for patients residing far from the trial site, the explanation and informed consent procedures described in 10.1.5 will be conducted using an online medical consultation system or similar methods.

10.1.8. Matters Concerning Protection of Subjects' Personal Information

All parties involved in this trial shall give due consideration to the protection of subjects' personal information and privacy in accordance with applicable laws and regulations.

Subjects shall be informed that monitors, auditors, the Institutional Review Board, and regulatory authorities may have access to source documents; such access shall be conducted while preserving the confidentiality of the subjects, and information obtained in this trial shall not be disclosed to third parties except when requested by public authority.

10.1.9. Establishment of Committees

Not applicable.

10.1.10. Data Quality Assurance

10.1.10.1. Trial Site

The principal investigator or sub-investigator shall complete case report forms for all subjects concerning screening, investigational product administration, and subsequent follow-up.

Case report forms for this trial shall be entered into an electronic data capture (EDC) system.

The principal investigator shall verify that the content of the case report forms is appropriate and shall apply an electronic signature.

The trial site shall retain as the case report form the case report form data generated in the EDC system (including the principal investigator's electronically signed records), audit trails, and electronic signature information stored on electronic media capable of long-term preservation.

Quality management at the trial site shall be performed in accordance with the site's own regulations.

The head of the trial site and the principal investigator shall provide direct access to all source documents and other trial-related records and shall cooperate during monitoring, auditing, regulatory inspections, and Institutional Review Board inquiries.

10.1.10.2. Monitoring

The monitor shall conduct monitoring in accordance with the trial's "Procedures for Conducting Monitoring" to confirm that the trial is performed in compliance with GCP, the clinical trial protocol, standard operating procedures, and applicable regulations, and that data reliability is adequately ensured.

Monitoring should include on-site direct review, such as source data verification comparing source documents with case report forms, as well as off-site monitoring.

If the monitor identifies any discrepancy between source documents and case report forms, the monitor shall obtain from the principal investigator a record explaining the reason for the discrepancy.

10.1.10.3. Data Management

The data management leader and staff shall implement quality control at each stage of data handling, in accordance with the "Procedures for Data Management Activities," to ensure the reliability of all trial-related data and its proper processing.

In addition, the procedures for EDC system development, case report form preparation, data review, and data lock shall be carried out in accordance with the Data Management Plan and related documents.

Upon completion of the data management activities, a "Data Management Report" will be prepared.

10.1.10.4. Audit

The auditor shall evaluate, independent of routine monitoring and clinical-trial quality-management activities, that the conduct of the trial, data generation, documentation (recording), and reporting are performed in compliance with GCP, the clinical trial protocol, standard operating procedures, and applicable regulations. The auditor shall conduct the audit from a third-party perspective as part of quality-assurance activities, in accordance with the trial's "Audit Plan" and the "Standard Operating Procedure for Conducting Audits."

10.1.10.5. Clinical Trial Coordinator

The Clinical Trial Coordinator shall be responsible for the quality of the trial data; therefore, he/she shall establish the trial's quality-control standards, implement a quality-management system, and assure quality based on a risk-based approach.

10.1.11. Identification of Source Data*

1) Source documents are evidence that the subject actually exists and that the collected data is complete. Source documents shall be retained at the trial site where the principal investigator is affiliated.

2) Data entered in the case report form on the basis of source documents must be consistent with the source documents; any discrepancy must be explained. As needed, the principal investigator or sub-investigator shall request retrieval or provision of prior medical records. In addition, the most recent medical records must be available for review.

3) The source documents that constitute the basis for the data are listed below. The trial site

shall prepare a list identifying the source documents.

- ✓ Records of subject consent and information provision to the subject (signed informed consent form, medical information release, etc.)
- ✓ Medical records, sealed charts or worksheets (documents created for this trial and attached to the medical record), nursing records, documents related to case enrollment, and the evaluation, test, and observation records specified in this trial (laboratory data, films, test requisitions), i.e., records that form the basis for CRF completion
- ✓ Records of the treatment administered in this trial (surgical records, etc.)

4) The handling of test and observation records performed at external (partner) sites is described in Appendix 4.

10.1.12. Costs and Conflict of Interest

10.1.12.1. Funding Source and Conflict of Interest

This trial will be conducted using research funding from the Japan Agency for Medical Research and Development (AMED), a national research and development corporation.
Conflicts of interest of individuals involved in this trial, such as the Clinical Trial Coordinator, principal investigator, and sub-investigators, shall be managed appropriately in accordance with the policies of the trial site. The conflict-of-interest status shall also be disclosed to subjects in the explanatory documents.

10.1.12.2. Costs Related to the Clinical Trial

Costs for medical care, surgery, examinations, and hospitalizations required for examinations during the clinical trial period shall be covered by health-insurance benefits. Monteplase (recombinant) will be purchased with research funds, and subjects will incur no charge.

10.1.12.3. Payment of Burden-Reduction Allowance

In accordance with the regulations of each trial site, a burden-reduction allowance shall be paid to subjects.

10.1.13. Initiation and Termination of the Trial at the Trial Sites

10.1.13.1. Initiation of the Trial

The date on which the investigational product is first delivered to the initial trial site shall be considered the trial start date.

10.1.13.2. Termination of the Trial at a Trial Site

After all subjects have completed the observations, examinations, and assessments stipulated in this clinical trial protocol, the principal investigator shall report in writing to the head of the trial site that the trial has been completed and provide a summary of the trial results. The head of the trial site shall promptly notify the Institutional Review Board in writing of the trial's termination and based on the report submitted by the principal investigator, shall report a summary of the trial results.

10.1.13.3. Suspension of the Trial

The clinical trial coordinator shall suspend the entire trial under any of the following circumstances:

1) When, based on matters concerning the quality, efficacy, or safety of the investigational product, the clinical trial coordinator judges that continuation of the trial is infeasible.

2) When, on the basis of the information in 1), the head of the trial site orders suspension.

3) When, for any other reason, the clinical trial coordinator determines that the trial should not continue.

If any of the above suspension criteria arise, the clinical trial coordinator and the principal investigator shall immediately confer regarding trial suspension or related actions. Upon making a decision to suspend the trial, the clinical trial coordinator shall promptly inform all trial personnel. The principal investigator shall promptly inform subjects of the suspension and its reasons, and shall ensure the safety of the subjects. The principal investigator shall report the suspension in writing to the head of the trial site, the Institutional Review Board, and the relevant departments of the affiliated medical institution, and shall follow the procedures established by that institution.

10.1.14. Policy Regarding Disclosure

Information on this trial will be made public through registration in the Japan Registry of Clinical Trials (jRCT). The results of this trial belong to Saga University. Publication of the results in conference presentations, journal articles, etc., requires the approval of the clinical trial coordinator. In addition, the data may be provided to pharmaceutical companies or other entities for purposes such as marketing-authorization applications.

10.1.15. Secondary Use of Data

Data collected in this trial may be used for purposes such as submission dossiers to regulatory authorities in Japan and abroad, and for research aimed at elucidating the mechanism of action,

among others. The explanatory document provided to subjects will include and describe, in addition to participation in this trial, the prospective secondary use of data. If consent to this trial is obtained, it shall be deemed that consent for the secondary use of data has also been obtained.

Appendix 2

10.2. Definition, Severity, and Causality of Adverse Events and Serious Adverse Events

10.2.1. Detailed Definition and Explanation of Adverse Events

<Definition of Adverse Event>

An adverse event (AE) is any unfavorable and unintended sign (including abnormal clinical laboratory values) that occurs in a subject temporally associated with the use of the investigational product, regardless of whether a causal relationship with the investigational product exists. A "side effect" refers to an AE for which a causal relationship with the investigational product is established. The criteria for causality assessment are described in 10.2.3. Recording and follow-up of Adverse Events and Serious Adverse Events.

<Examples Considered as Adverse Events>

- ✓ Abnormalities in clinical laboratory values (hematology, clinical chemistry, urinalysis) or other safety-assessment parameters (including worsening from baseline) that, in the judgment of the principal investigator or sub-investigator based on medical and scientific assessment, are clinically significant (i.e., not related to progression of the underlying disease or represent a deterioration of the subject's condition beyond what was anticipated). Clinical significance includes:
    - Presence of associated symptoms
    - Requirement for additional investigations, or need for medical or surgical treatment
    - Discontinuation of the trial, or addition of clinically meaningful concomitant medication or other therapy
- ✓ Exacerbation of chronic or intermittent symptoms that were present before trial initiation (including increased frequency and/or severity of symptoms).
- ✓ Newly identified or diagnosed symptoms after the start of investigational product administration (even if the symptom may have existed prior to trial initiation, it is considered an AE if identified or diagnosed after dosing).
- ✓ Signs, symptoms, or sequelae suggestive of a drug-drug interaction.
- ✓ Signs, symptoms, or sequelae suggestive of overdose of the investigational product or concomitant medication. Overdose itself is not reported as an AE or SAE, except that intentional overdose performed with the intent of suicide or self-harm must be reported regardless of the presence of sequelae.

<Examples Not Considered as Adverse Events>

- ✓ Abnormalities in clinical laboratory values or fluctuations in other safety-assessment parameters that are related to the underlying disease, except when the principal investigator or sub-investigator judges that the subject's condition is clinically more severe than expected.
- ✓ Progression of the disease under study and the emergence of new lesions per se are not regarded as AEs. However, worsening of symptoms associated with the underlying disease is treated as an AE.
- ✓ Planned medical or surgical procedures (e.g., endoscopic examinations).
- ✓ Unfavorable medical occurrences that have not manifested (e.g., social or convenience hospitalizations).
- ✓ Variations in diseases or conditions that were confirmed or identified before trial initiation and that fall within the expected daily variability or that have not worsened.
- ✓ The lack of efficacy or the absence of the expected pharmacologic effect is not reported as an AE. These examples are described in the efficacy evaluation.

10.2.2. Definition of Serious Adverse Events

A serious adverse event (SAE) is any untoward medical occurrence, regardless of dose, that meets any of the following criteria.

a. Resulting in death.
b. Life-threatening.
"Life-threatening" means that, at the time the event occurred, the subject was at risk of death; it does not imply a hypothetical situation in which a more severe manifestation of the event might have caused death.
c. Events that require hospitalization or prolongation of an existing hospitalization for treatment.

- ✓ In general, hospitalization refers to the subject staying in a hospital or emergency department (typically overnight or longer) to receive observation and/or treatment that cannot be appropriately performed in a clinic or outpatient setting. Complications that arise during hospitalization are considered adverse events; if the complication results in an extension of the hospital stay, or if the complication itself meets another SAE criterion, it shall be classified as an SAE.
- ✓ Hospitalization for treatment of a comorbidity that has not worsened since the start of the clinical trial, hospitalization for administration of the investigational product, hospitalization for examinations, or hospitalization intended to reduce the burden on

subjects traveling from distant locations shall not be considered a SAE.

d. Results in persistence or significant disability or incapacity.

- ✓ Refers to a condition that markedly impairs the subject's normal daily functioning.
- ✓ Medical events that are not of substantial importance, such as headache, nausea, vomiting, diarrhea, influenza, or minor injuries (e.g., ankle sprain) without accompanying complications, are excluded.

e. Congenital anomaly or birth defect.

f. Other medically important conditions:

- ✓ Although the event does not immediately threaten life, result in death, or require hospitalization, if it constitutes a serious medical occurrence that places the subject at risk or necessitates medical or surgical intervention to prevent the outcomes listed above, the principal investigator or sub-investigator shall determine, based on medical and scientific justification, whether reporting as an SAE is required.
  Examples include infiltrative or malignant cancer, allergic bronchospasm requiring intensive care in an emergency department or at home, and hematologic disorders or seizures that do not necessitate hospitalization.

10.2.3. Recording and follow-up of Adverse Events and Serious Adverse Events

<Recording of Adverse Events and Serious Adverse Events>

The principal investigator or sub-investigator at the trial site shall be responsible for evaluating all records related to an AE/SAE that occur during the trial period (e.g., hospital progress notes, clinical laboratory records, diagnostic reports).

The principal investigator or sub-investigator shall document the following information concerning each AE/SAE in the source documents.

[Items]

- Adverse event name
- Onset date
- Severity classification (mild / moderate / severe)
- Seriousness classification (serious / non-serious)
- Causality assessment (no relationship / relationship present)
- Outcome (recovered / improved / not recovered (unchanged) / recovered with sequelae / death / unknown)
- Outcome date
- Management (whether treatment was given and its details)

The principal investigator or sub-investigator shall, based on signs, symptoms, and/or other clinical information, make every effort to identify the diagnostic term for the event. When possible, the diagnostic term—not individual signs or symptoms—should be recorded as an AE/SAE.

<Severity Assessment>

The principal investigator or sub-investigator shall classify the severity of an adverse event according to the following definitions:

Mild: Discomfort is present but does not interfere with daily activities.

Moderate: Some interference with daily activities is observed.

Severe: The subject is unable to carry out daily activities or work.

<Causality Assessment>

The principal investigator or sub-investigator shall evaluate the relationship between the investigational product and each AE/SAE and determine clinically whether a causal relationship is "present" or "absent."

Causality determinations shall be made as follows:

- ✓ No relationship

  The event can be clearly explained by comorbidities, medical history, concomitant medications, the known pharmacologic properties of the investigational product, or other known causes such as surgery.
- ✓ Relationship present (all three criteria must be satisfied)
  - i. The temporal relationship between the onset of the event and the administration of the investigational product can be reasonably explained.
  - ii. The event could plausibly arise from the pharmacologic action, physicochemical properties, route of administration, or other known characteristics of the investigational product or similar agents, or the principal investigator/sub-investigator acknowledges a causal relationship.
  - iii. The subject's comorbidities, medical history, concomitant drugs, or concomitant therapies are not considered contributory.

In performing the causality assessment, the principal investigator or sub-investigator shall refer to the Investigator's Brochure, the product label, and the interview form.

In certain situations, when an SAE occurs, the principal investigator may not have obtained all information required for the initial report to the trial coordinator. Nevertheless, the principal investigator must conduct a causality assessment for each event before transmitting the SAE

data to the trial coordinator as the first report. The principal investigator or sub-investigator may revise the causality opinion in light of additional information, amend the causality assessment accordingly, and prepare an updated SAE follow-up report.

<Outcome>

The outcome of an adverse event shall be classified into one of the following categories.

1) Recovered: The adverse event has resolved, and the subject has returned to the pre-onset condition.

2) Improving: The adverse event has almost resolved, and the subject has returned to a condition close to that before onset.

3) Not recovered: The adverse event has not resolved, and the subject remains in the same condition as at the time of onset (unchanged).

4) Recovered with sequelae: The adverse event has resolved, but the subject has residual sequelae.

5) Death: The subject has died (regardless of causality).

6) Unknown: Outcome is unknown due to lack of information.

<Follow-up of Adverse Events and Serious Adverse Events>

The principal investigator or sub-investigator shall, when medically necessary or upon request from the trial coordinating physician, conduct or arrange additional examinations and/or assessments as needed to elucidate, to the greatest extent possible, the nature and/or causality of an AE or SAE. Such additional examinations may include clinical laboratory tests, ophthalmologic examinations, or consultations with other healthcare specialists.

Even if the subject does not return to normal or to the pre-investigational-product level, the principal investigator or sub-investigator may discontinue follow-up for that subject if, based on the follow-up findings, they determine that further follow-up is unnecessary and that such a decision is justified from the perspective of subject protection.

The principal investigator or sub-investigator shall document the information related to the follow-up.

10.2.4. Reporting of Serious Adverse Events

If an SAE occurs in this clinical trial, the principal investigator or sub-investigator shall, in accordance with the protocol's "Procedures for Handling Safety Information," promptly report the SAE to the head of the trial site, the trial coordinating physician, and the safety-information management officer. The trial coordinating physician shall immediately forward the report to the principal investigators at the other trial sites.

The principal investigator shall promptly report, for any SAE requiring follow-up, the information obtained from the follow-up to the head of the trial site, the trial coordinating physician, and the safety-information management officer. The trial coordinating physician shall also report such information to the principal investigators at the other trial sites.

<Contact for Serious Adverse Events>

- Coordinating Investigator
- Kyushu University Hospital ARO Next-Generation Medical Center Safety Information Management Unit

The trial coordinating physician shall discuss with the principal investigator whether the SAE falls under a 7-day or 15-day reporting requirement (Article 273, Paragraph 1, Items 1 and 2 of the Ordinance for Enforcement of the Pharmaceuticals and Medical Devices Act) and make a determination. If reporting is deemed necessary, the trial coordinating physician shall submit the report to the Pharmaceuticals and Medical Devices Agency (PMDA) in accordance with the "Procedures for Handling Safety Information." The trial coordinating physician shall promptly inform all principal investigators of any adverse event reported to the PMDA. In addition, the principal investigator shall report the content to the head of the trial site.

If the principal investigator receives instructions from the head of the trial site concerning amendments to the clinical trial protocol, based on the Institutional Review Board's opinion regarding the appropriateness of continuing the trial, the principal investigator shall comply with such instructions.

10.2.5. Collection of Safety Information

The clinical trial coordinator shall independently collect information on the quality, efficacy, and safety of the investigational product. The collected information shall be handled in accordance with the "Procedures for the Handling of Safety Information," and the necessary actions shall be taken.

Appendix 3

10.3. Guidance on Contraception

10.3.1. Definition of Women of Child-bearing Potential

Women participants of child-bearing potential

A subject is considered to be of child-bearing potential if she meets any of the following criteria:
1. She has experienced menarche.
2. She has not undergone a sterilization procedure and has not reached a post-menopausal state.

- ✓ Post-menopausal state* is defined as the absence of menstruation for ≥ 12 months in the absence of any other medical cause.
- ✓ Serum follicle-stimulating hormone (FSH) levels may be used to confirm post-menopausal status when the subject is not receiving hormonal contraception or hormone replacement therapy (HRT). If the amenorrhea period is < 12 months, confirmation by ≥ two separate FSH measurements is desirable.
- ✓ If the subject is receiving HRT and her menopausal status is uncertain, continuation of HRT during the trial is permissible only if she uses a highly effective, non-estrogenic contraceptive method. Otherwise, HRT must be discontinued prior to trial enrollment in order to verify post-menopausal status.
- ✓ Sterilization procedures include hysterectomy, bilateral salpingectomy, and bilateral oophorectomy. For subjects with permanent infertility due to medical conditions (e.g., Müllerian agenesis, androgen insensitivity syndrome, gonadal dysgenesis), participation in the trial shall be determined by the principal investigator.

10.3.2. Contraception Guidance

For female subjects of child-bearing potential, from the time of obtaining informed consent until 2 days after the last dose of the investigational product, the subject must either abstain from heterosexual intercourse or employ an effective contraceptive method such as barrier protection (condom, pessary*, cervical cap*), hormonal intra-uterine device, copper intra-uterine device, etc. (*These devices are not approved or certified for use in Japan.)

Appendix 4

10.4. Implementation at Partner Sites

In this clinical trial, a portion of subject visits may be conducted at partner sites that meet the following requirements. Nevertheless, the principal investigator or sub-investigator of the implementing medical institution retains responsibility for all trial-related activities performed at the partner site.

<Selection Criteria for Partner Medical Institutions>
A medical institution that satisfies all of items ①−③ below shall be selected as a partner medical institution and a contract shall be executed with the implementing medical institution.

① At least one physician permanently stationed at the institution holds the qualification of a Japanese Ophthalmological Society specialist.
② Medical devices used for observations during the trial period are subject to regular inspection and precision management.
③ An internet environment and telemedicine equipment (including a web camera of sufficient resolution to verify test results) capable of connecting with the implementing medical institution via a remote-consultation system are in place.

<Activities That May Be Performed When a Subject Visits a Partner Medical Institution>
- ✓ Explanation of the trial and obtaining informed consent
- ✓ Screening
- ✓ Assessments at Visit 6 (Week 2/Day 15), Visit 7 (Week 3/Day 22) and Visit 9 (Week 8/Day 57)
- ✓ Assessments at discontinuation
- ✓ Unscheduled visits (when deemed permissible by the principal investigator or sub-investigator)

<Receipt of Test Data from the Partner Site>
On the day of the visit, the trial site and the partner site shall be linked via the remote-consultation system, and the principal investigator or sub-investigator of the trial site shall confirm the test results performed at the partner site through a web camera.
After the remote consultation is completed, the physician at the partner site shall date and sign the printed test results and send them to the trial site as a certified copy by postal mail. In

principle, monitoring will be performed on the certified copy sent to the trial site; however, the partner site must respond to any inquiries from the monitor as necessary.

Appendix 5

10.5. Preservation of Records

10.5.1. Trial Sites

The record-custodian appointed by the head of the trial site shall retain the essential documents required by GCP (including copies and records) at the location designated by the trial site.

The retention period shall be until the later of the following dates. However, if the principal investigator requires a longer retention period, the period and method shall be discussed. The principal investigator shall notify the head of the trial site when the documents are no longer required to be retained.

- ✓ The date marketing authorization is obtained (or, if development is discontinued or a notice is received that the results of this trial will not be attached to the marketing-application dossier, three years after the date of such notice)
- ✓ The date three years after the discontinuation or completion of this clinical trial

If disposal is to occur after the retention period expires, prior approval from the trial-coordinating physician shall be obtained.

If the storage location is to be changed for any reason, appropriate measures should be taken to prevent loss or disposal of the records during the retention period. Where necessary, a record of the relocation shall be maintained under the responsibility of the head of the institution or the record-custodian.

10.5.2. Institutional Review Board

The sponsor of the Institutional Review Board shall retain the documents that, according to GCP, must be kept by the IRB.

The retention period shall be until the later of the following dates. However, if the principal investigator requires a longer retention period, the period and method shall be discussed.

- ✓ The date on which the investigational medicinal product provider or its development partner continuing the development obtains marketing authorization (or three years have elapsed from the date of receipt of notification of development discontinuation or notification that the results of this study will not be included in the marketing authorization application, whichever is earlier).
- ✓ The date is three years after the discontinuation or completion of this clinical trial.

The principal investigator shall notify the IRB sponsor when the documents are no longer

required to be retained.

10.5.3. Sponsor-Investigator and Trial Coordinating Physician

The sponsor-investigator and the trial-coordinating physician shall retain, among the documents required by GCP, those that the sponsor-investigator is obligated to keep.

The location and duration of retention shall be in accordance with the “Procedures for Record Retention” applicable to the trial.

The trial-coordinating physician shall notify the sponsor-investigator (principal investigator) when the documents are no longer required to be retained.

10.5.4. Preservation of Records at Other Institutions

Contract research organizations, site management organizations, central laboratories, and partner sites involved in this clinical trial shall retain the contract and the documents required to be archived under the contract until the later of the following dates. ✓ the approval date of the investigational product for the clinical trial

✓ the date that is three years after the discontinuation or completion of the clinical trial

Appendix 6

10.6. Revision History of the Clinical Trial Protocol

Date	Version	Reason for Revision
24 April 2025	Version 1.1	Refer to the new-old comparison table
28 July 2025	Version 1.2	Refer to the new-old comparison table
26 September 2025	Version 1.3	Refer to the new-old comparison table
17 December 2025	Version 1.4	Refer to the new-old comparison table

11. References

1. Hochman M, A., Seery C, M. and Zarbin M, A. Pathophysiology and management of subretinal hemorrhage. Surv Ophthalmol 1997; 42: 195-213. DOI: 10.1016/s0039-6257(97)00089-1.
2. Scupola A, Coscas G, Soubrane G, et al. Natural history of macular subretinal hemorrhage in age - related macular degeneration. Ophthalmologica 1999; 213: 97 - 102. DOI: 10.1159/000027400.
3. Al-Hity A, Steel DH, Yorston D, et al. Incidence of submacular haemorrhage (SMH) in Scotland: a Scottish Ophthalmic Surveillance Unit (SOSU) study. Eye (Lond) 2019; 33: 486-491. 20181029. DOI: 10.1038/s41433-018-0239-4.
4. McGowan G, F., Steel D and Yorston D. AMD with submacular hemorrhage: new insights from a population-based study. ARVO Annual Meeting Abstract. 2014.
5. Kimura T, Araki T, Yasukawa T, et al. Differences in clinical characteristics and treatment outcomes of submacular hemorrhage caused by age-related macular degeneration and retinal macroaneurysms: a multicenter survey from the Japan Clinical Retina Study (J-CREST) group. PLoS One 2022; 17: e0274508. 20220929. DOI: 10.1371/journal.pone.0274508.
6. Gabrielle PH, Maitrias S, Nguyen V, et al. Incidence, risk factors and outcomes of submacular haemorrhage with loss of vision in neovascular age-related macular degeneration in daily clinical practice: data from the FRB! registry. Acta Ophthalmologica 2022; 100. DOI: 10.1111/aos.15137.
7. Morizane Y, Morimoto N, Fujiwara A, et al. Incidence and causes of visual impairment in Japan: the first nation-wide complete enumeration survey of newly certified visually impaired individuals. Jpn J Ophthalmol 2019; 63: 26-33. 20180925. DOI: 10.1007/s10384-018-0623-4.
8. Wong WL, Su X, Li X, et al. Global prevalence of age-related macular degeneration and disease burden projection for 2020 and 2040: a systematic review and meta-analysis. Lancet Glob Health 2014; 2: e106-116. 20140103. DOI: 10.1016/S2214-109X(13)70145-1.
9. Kido A, Miyake M, Tamura H, et al. Incidence and clinical practice of exudative age-related macular degeneration: a nationwide population-based cohort study. Ophthalmol Sci 2022; 2: 100125. 20220301. DOI: 10.1016/j.xops.2022.100125.
10. Rabb M, F., Gagliano D, A. and Teske M, P. Retinal arterial macroaneurysms. Surv Ophthalmol 1988; 33: 73-96.
11. Tonotsuka T, Imai M, Saito K, et al. Visual prognosis for symptomatic retinal arterial macroaneurysm. Jpn J Ophthalmol 2003; 47: 498-502. DOI: 10.1016/s0021-5155(03)00104-7.
12. Benntee S, R., Folk J, C., Boldi C, F., et al. Factors prognostic of visual outcome in patients

with subretinal hemorrhage. Am J Ophthalmol 1990; 109: 33-37.

13. Glatt H and Machemer R. Experimental subretinal hemorrhage in rabbits. Am J Ophthalmol 1982; 94: 762-773.

14. Lu A, Q., Prensky J, G., Baker P, S., et al. Update on medical and surgical management of submacular hemorrhage. Expert Rev Ophthalmol 2020; 15: 43-57.

15. Lim JI, Drews-Botsch C, Sternberg P, Jr., et al. Submacular hemorrhage removal. Ophthalmology 1995; 102: 1393-1399. DOI: 10.1016/s0161-6420(95)30858-5.

16. Toth C, A., Morse LS, Hjelmeland L, M., et al. Fibrin directs early retinal damage after experimental subretinal hemorrhage. Acta Ophthalmologica 1991; 109: 723-729.

17. Stanescu-Segall D, Balta F and Jackson TL. Submacular hemorrhage in neovascular age-related macular degeneration: A synthesis of the literature. Surv Ophthalmol 2016; 61: 18-32. 20150723. DOI: 10.1016/j.survophthal.2015.04.004.

18. Jackson TL, Bunce C, Desai R, et al. Vitrectomy, subretinal tissue plasminogen activator and intravitreal gas for submacular haemorrhage secondary to exudative age-related macular degeneration (TIGER): study protocol for a phase 3, pan-European, two-group, non-commercial, active-control, observer-masked, superiority, randomised controlled surgical trial. Trials 2022; 23: 99. 20220131. DOI: 10.1186/s13063-021-05966-3.

19. Miki M, Miyata M, Ooto S, et al. Predictors of 3-month and 1-year visual outcomes after vitrectomy with subretinal tissue plasminogen activator injection for submacular hemorrhage. Retina 2023; 43: 1971-1979. DOI: 10.1097/IAE.0000000000003885.

20. Fukuda Y, Nakao S, Kohno RI, et al. Postoperative follow-up of submacular hemorrhage displacement treated with vitrectomy and subretinal injection of tissue plasminogen activator: ultrawide-field fundus autofluorescence imaging in gas-filled eyes. Jpn J Ophthalmol 2022; 66: 264-270. 20220309. DOI: 10.1007/s10384-022-00910-7.

21. Kawakami S, Wakabayashi Y, Umazume K, et al. Long-term outcome of eyes with vitrectomy for submacular and/or vitreous hemorrhage in neovascular age-related macular degeneration. J Ophthalmol 2021; 2021: 2963822. 20211102. DOI: 10.1155/2021/2963822.

22. Kimura S, Morizane Y, Hosokawa M, et al. Outcomes of vitrectomy combined with subretinal tissue plasminogen activator injection for submacular hemorrhage associated with polypoidal choroidal vasculopathy. Jpn J Ophthalmol 2019; 63: 382-388. DOI: 10.1007/s10384-019-00679-2.

23. Kimura M, Yasukawa T, Shibata Y, et al. Flattening of retinal pigment epithelial detachments after pneumatic displacement of submacular hemorrhages secondary to age-related macular degeneration. Graefe’s Archive for Clinical and Experimental Ophthalmology 2018; 256: 1823-1829. DOI: 10.1007/s00417-018-4059-9.

24. Kadonosono K, Arakawa A, Yamane S, et al. Displacement of submacular hemorrhages in

age-related macular degeneration with subretinal tissue plasminogen activator and air. Ophthalmology 2015; 122: 123-128. 20140904. DOI: 10.1016/j.ophtha.2014.07.027.
25. Kimura S, Morizane Y, Hosokawa M, et al. Submacular hemorrhage in polypoidal choroidal vasculopathy treated by vitrectomy and subretinal tissue plasminogen activator. American Journal of Ophthalmology 2015; 159: 683-689.e681. DOI: 10.1016/j.ajo.2014.12.020.
26. Fujikawa M, Sawada O, Miyake T, et al. Comparison of pneumatic displacement for submacular hemorrhages with gas alone and gas plus tissue plasminogen activator. Retina 2013; 33: 1908-1914. DOI: 10.1097/IAE.0b013e318287d99d.
27. Mizutani T, Yasukawa T, Ito Y, et al. Pneumatic displacement of submacular hemorrhage with or without tissue plasminogen activator. Graefe's Archive for Clinical and Experimental Ophthalmology 2011; 249: 1153-1157. 20110329. DOI: 10.1007/s00417-011-1649-1.
28. Terasaki H, Miyake Y, Kondo M, et al. Focal macular electroretinogram before and after drainage of macular subretinal hemorrhage. American Journal of Ophthalmology 1997; 123: 207-211. DOI: 10.1016/s0002-9394(14)71037-2.
29. Kamei M, Tano Y, Maeno T, et al. Surgical removal of submacular hemorrhage using tissue plasminogen activator and perfluorocarbon liquid. Am J Ophthalmol 1996; 121: 267-275. DOI: 10.1016/s0002-9394(14)70274-0.
30. Tsuiki E, Kusano M and Kitaoka T. Complication associated with intravitreal injection of tissue plasminogen activator for treatment of submacular hemorrhage due to rupture of retinal arterial macroaneurysm. American Journal of Ophthalmology Case Reports 2019; 16. DOI: 10.1016/j.ajoc.2019.100556.
31. Inoue M, Shiraga F, Shirakata Y, et al. Subretinal injection of recombinant tissue plasminogen activator for submacular hemorrhage associated with ruptured retinal arterial macroaneurysm. Graefes Arch Clin Exp Ophthalmol 2015; 253: 1663-1669. 20141125. DOI: 10.1007/s00417-014-2861-6.
32. Chen SN, Yang TC, Ho CL, et al. Retinal toxicity of intravitreal tissue plasminogen activator: case report and literature review. Ophthalmology 2003; 110: 704-708. DOI: 10.1016/S0161-6420(02)01979-6.
